# Supplementary material for: Sub-femtomolar drug monitoring via co-calibration mechanism with nanoconfined DNA probes
Source: Nat Commun. 2025 Feb 21;16:1863. doi: 10.1038/s41467-025-57112-1 (PMC11845792; doi:10.1038/s41467-025-57112-1)
Supplement: Supplementary file 1 — Supplementary Information [file 41467_2025_57112_MOESM1_ESM.pdf]

Supplementary Information for

Sub-femtomolar drug monitoring via co-calibration mechanism with nanoconfined DNA probes

Yonghuan Chen[1,2], Xiuying Li[1], Xinru Yue[1], Weihua Yu[1], Yuesen Shi[3], Zilong He[1], Yuanfeng Wang[4], Yu Huang[2]*, Fan Xia[2], and Fengyu Li[1,5]*

[1] College of Chemistry and Materials Science, Guangdong Provincial Key Laboratory of Speed Capability Research, Su Bingtian Center for Speed Research and Training, Jinan University, Guangzhou 510632, China.

[2] State Key Laboratory of Biogeology and Environmental Geology, Engineering Research Center of Nano-Geomaterials of Ministry of Education, Faculty of Material Science and Chemistry, China University of Geosciences, Wuhan 430074, China.

[3] Anti-Drug Technology Center of Guangdong Province, Guangdong Province Key Laboratory of Psychoactive Substances Monitoring and Safety, Guangzhou 510230, China.

[4] Key Laboratory of Evidence Science, China University of Political Science and Law, Beijing 100088, China.

[5] College of Chemistry, Zhengzhou University, 450001 Zhengzhou, China.

[*] Correspondence and requests for materials should be addressed to F.L. (email: lifengyu@jnu.edu.cn) or to Y.H. (email: yuhuang@cug.edu.cn).

This file includes:

Supplementary Methods

Supplementary Figures 1 to 36

Supplementary Tables 1 to 14

Supplementary References

Table of Contents

1. Supplementary Methods

1.1. Fabrication of CBA@AAO N_CB_S.
1.2. Ultra-high resolution scanning electron microscope (SEM).
1.3. Energy dispersive spectrometer (EDS).
1.4. X-ray photoelectron spectroscopy (XPS) measurements.

2. Supplementary Figures. 1-36

Supplementary Figure 1. CA of DNA probes-N_CB_S via varying CBA:C4 DNA ratios.
Supplementary Figure 2. XPS of CBA@AAO and CBA&C4@AAO.
Supplementary Figure 3. Fabrication of the solid-state N_CB_S functionalized with DNA probe.
Supplementary Figure 4. AAO and GA@AAO nanochannel membrane diagram.
Supplementary Figure 5. Morphological and TmIC of different nanochannels.
Supplementary Figure 6. SEM characterization and pore distribution analysis of nanochannels.
Supplementary Figure 7. EDS mapping of nanochannel membranes.
Supplementary Figure 8. XPS of the surfaces of nanochannel membranes.
Supplementary Figure 9. CA test diagram.
Supplementary Figure 10. Parallel experiments of five groups of CA test.
Supplementary Figure 11. TmIC changes of GA@AAO and AAO upon CBA incubation.
Supplementary Figure 12. Specific fabrication of CBA@AAO.
Supplementary Figure 13. TmIC changes of AAO and GA@AAO with various CBA.
Supplementary Figure 14. Optimization of CBA incubation time.
Supplementary Figure 15. Comparison of current magnitude and ionic current signals for N_CB_S.
Supplementary Figure 16. PolyA@AAO and CBA@AAO N_CB_S sensitivity.
Supplementary Figure 17. Cycling of CBA@AAO for 1 pM cathinone direct detection.
Supplementary Figure 18. Four different sizes of AAO nanochannel membranes.
Supplementary Figure 19. TmIC response of CBA@AAO with different cathinone concentrations.
Supplementary Figure 20. *I-T* measurements of CBA@AAO with cathinone at +2 V.
Supplementary Figure 21. Structures of the target analyte and 13 psychoactive drugs.
Supplementary Figure 22. Selectivity of the CBA@AAO.
Supplementary Figure 23. *I-V* of CBA@AAO with target and 13 psychoactive drugs.
Supplementary Figure 24. Principal component analysis (PCA) score plot for distinguishing different concentrations of Cat, Met, and Eth.
Supplementary Figure 25. PCA score plot was utilized to differentiate 14 different drug analytes.
Supplementary Figure 26. pH-responsive C4@AAO.
Supplementary Figure 27. TmIC changes of N_CB_S with varying CBA:C4 DNA ratios.
Supplementary Figure 28. Current changes of N_CB_S modified with different proportions of DNA molecules before and after response to 1 pM cathinone.
Supplementary Figure 29. TmIC responses of CBA&C4@AAO and its dependence on pH and target concentration.
Supplementary Figure 30. CD and UV spectral responses of CBA and PolyA upon interaction with cathinone and its analogs.

Supplementary Figure 31. Molecular dynamics cyclotron radius diagram of the complex.
Supplementary Figure 32. Average solvent-accessible and total surface area of protein residues in the experimental system.
Supplementary Figure 33. Schematic diagram of co-calibration of cathinone and pH with dual-DNA probes N_CB_S.
Supplementary Figure 34. TmIC variation of CBA&C4@AAO under different conditions.
Supplementary Figure 35. Orthogonal responsiveness of CBA&C4@AAO.
Supplementary Figure 36. Spike-and-recovery experience.

3. Supplementary Tables 1-14

Supplementary Table 1. Sequences of all single-stranded DNA oligonucleotides.
Supplementary Table 2. XPS date of the CBA&C4@AAO before and after preparation.
Supplementary Table 3. Pore diameter, density and porosity for nanochannels.
Supplementary Table 4. XPS date of the AAO.
Supplementary Table 5. XPS date of the GA@AAO.
Supplementary Table 6. XPS date of the CBA@AAO.
Supplementary Table 7. XPS narrow spectra date of the AAO.
Supplementary Table 8. XPS narrow spectra date of the GA@AAO.
Supplementary Table 9. XPS narrow spectra date of the CBA@AAO.
Supplementary Table 10. Pore diameter, density and porosity of five types of AAO.
Supplementary Table 11. Jackknifed classification matrix of 3 drugs.
Supplementary Table 12. Jackknifed classification matrix of 14 drugs at identical concentrations.
Supplementary Table 13. Jackknife classification matrix of 4 drugs and two disruptors in artifical sweat.
Supplementary Table 14. Research progress on cathinone monitoring.

4. Supplementary References 1-10

1. Supplementary Methods

1.1. Fabrication of CBA@AAO N_CB_S.

The design strategy for the solid-state N_CB_S functionalized with DNA probes is shown in Supplementary Figure 3a. The anodic aluminum oxide (AAO) nanochannel was modified to an aldehyde group (GA@AAO) through a two-step chemical reaction. Subsequently, a 5'-amino-modified cathinone-specific binding DNA probe (CBA) was grafted onto the AAO via a Schiff base reaction, thus functionalizing it (CBA@AAO). The SEM images of the nanochannel membrane illustrate the changes in effective pore diameter before and after functionalization (Supplementary Figure 6). The bare AAO exhibits an orderly channel structure with a pore diameter distribution of 60.3±7.0 nm, whereas CBA@AAO shows a disordered surface and a reduced effective pore diameter of 49.9±7.5 nm (Supplementary Figures 3b, c). To confirm the successful grafting of CBA and its response to cathinone, CA measurements were used to characterize the surface of the modified AAO membrane. The CA values of the AAO in its original state, GA@AAO, CBA@AAO, and CBA@AAO bound to cathinone were 51.7±0.4°, 57.7±0.2°, 55.1±0.7°, and 60.9±0.4°, respectively (Supplementary Figures 3d, 9). All these changes in wettability of the AAO correspond to changes in chemical composition.

The successful fabrication of CBA@AAO can be corroborated by measuring the changes in ionic current through the nanochannels before and after modification, which reflect changes in effective diameter and surface wettability. As shown in Supplementary Figure 3e, the AAO exhibits a higher transmembrane ionic current (TmIC) due to the synergistic effect of effective size and surface wettability. In contrast, GA@AAO shows a corresponding decrease in TmIC due to the reduced effective diameter and increased surface hydrophobicity, with a signal change of approximately 35%. Although the effective diameter of CBA@AAO significantly decreases, its porosity increases compared to GA@AAO (Supplementary Table 3), and the introduction of the DNA probe enhances the hydrophilicity of the membrane surface. The combined competitive effects of surface wettability and size result in an increase in TmIC, with a significant signal change of approximately 57%. Supplementary Fig. 3f shows the EDS mapping of CBA@AAO before and after fabrication, qualitatively confirming N_CB_S functionalization through changes in nitrogen content (Supplementary Figure 7). Additionally, to further validate the successful grafting of CBA onto the

AAO membrane surface, XPS was used to quantitatively characterize the changes in nitrogen content before and after modification (Supplementary Tables 4-9 and Supplementary Figure 8). Unlike the unmodified membrane, the GA@AAO and CBA@AAO show characteristic N_{1s} XPS peaks at approximately 400 eV, indicating the successful immobilization of the probe on the membrane surface (Supplementary Figure 3g). The nitrogen content before and after modification was 1.03%, 4.49%, and 5.50%, respectively. These results provide substantial evidence for the successful binding of the cathinone-specific probe to the AAO membrane surface.

1.2. Ultra-high resolution scanning electron microscope (SEM).

To confirm the successful fabrication of the CBA@AAO, we conducted a detailed study of the surface morphology of AAO, GA@AAO, and CBA@AAO using SEM (Supplementary Table 3). The purchased AAO nanochannel membranes had a pore size distribution ranging from 40 to 70 nm. Statistical analysis revealed that the actual pore size distribution ranged from 48.1 to 88.3 nm, with an average pore size of 60.3±7.0 nm and a porosity of 17.5%. Compared to the bare AAO membrane, the pore size of the GA@AAO decreased, with a distribution between 36.8 and 83.5 nm, an average pore size of 56.4±8.1 nm, and a porosity of 18.9%. Notably, after introducing CBA, the pore size further decreased compared to GA@AAO, with an average pore size reduced to 49.9±7.5 nm, ranging from 34.3 to 77.6 nm, and the porosity increased to 21.6%.

1.3. Energy dispersive spectrometer (EDS).

GA@AAO compared with AAO, nitrogen and silicon on the membrane surface increase directly due to the introduction of APTES. With the continuous grafting of APTES and GA to the AAO membrane surface, the oxygen element on the surface is covered and slightly reduced. The successful preparation of CBA@AAO indicates that the content of nitrogen and carbon increases after the introduction of CBA (5'-terminal amination) to GA@AAO. Therefore, the changes of C, N, O and Si elements on AAO, GA@AAO and CBA@AAO membrane surfaces were observed by EDS mapping, which can roughly reflect the modification process (Supplementary Figure 7).

1.4. X-ray photoelectron spectroscopy (XPS) measurements.

XPS analysis was utilized to detect the content of nitrogen in order to confirm the DNA immobilized on AAO surface treated by Tris-HCl solution after modification (pH=7.4, 23 ℃). XPS was conducted on a Thermo Scientific™ K-Alpha™+ spectrometer equipped with a monochromatic Al Kα X-ray source (1486.6 eV) operating at 100 W. Samples were analyzed under vacuum ($P<10^{-8}$ mbar) with a pass energy of 150 eV (survey scans) or 50 eV (high-resolution scans). All peaks would be calibrated with C_{1s} peak binding energy at 284.8 eV for adventitious carbon.

In this work, due to the abundant -OH on the surface of AAO membrane, APTES can be grafted to the surface of AAO membrane by silanization through a two-step chemical modification method. The surface of the membrane was modified to $-NH_2$, and then the surface functional group was changed to -CHO by Schiff base reaction with GA. Subsequently, the 5'-amino modified CBA and GA@AAO were functionalized into the nanochannel by Schiff base reaction again. In order to verify the successful grafting of DNA probes onto AAO nanochannel membranes as specific sensing probes, the elemental compositions of AAO, GA@AAO and CBA@AAO membranes were analyzed by XPS.

Similar to the EDS analysis results, the relative content of nitrogen in GA@AAO nanochannels increased from 1.03% to 4.49%. After the successful grafting of CBA onto the AAO nanochannel membrane, the nitrogen content further increased to 5.50% (Supplementary Tables 4-9). As shown in Supplementary Figure 8a, the N_{1s} peak is significantly pronounced and shows an increasing trend. After functionalizing the AAO nanochannel membrane with CBA, the intensities of the C_{1s} (284.8 eV) and N_{1s} (~ 398 eV) peaks increased significantly, indicating an increase in the content of carbon and nitrogen elements (Supplementary Figures 8b, c). The content of oxygen elements showed an overall decreasing trend, particularly during the transition from AAO to GA@AAO. This decrease is due to the introduction of APTES and GA onto the nanochannel membrane, leading to the coverage of oxygen elements originally present on the AAO membrane (Supplementary Figure 8d). These XPS results further confirm the successful preparation of CBA@AAO.

2. Supplementary Figures

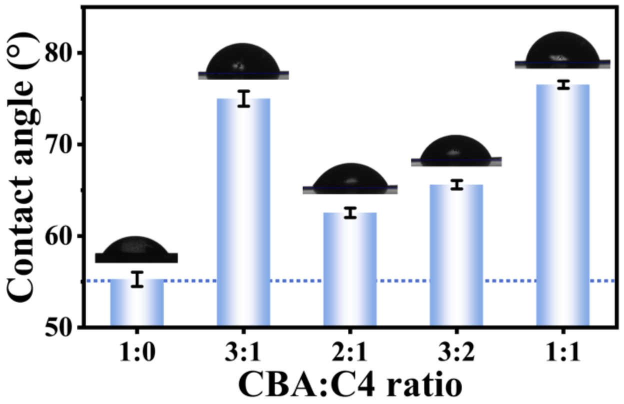

Supplementary Figure 1. CA of DNA probes-N_CB_S via varying CBA:C4 DNA ratios. This figure shows the effect of different CBA:C4 DNA probe mixing ratios on the contact angle of the functionalized nanopore membranes. The variation in contact angle reflects the modification of the membrane surface's hydrophilicity or hydrophobicity by DNA probe functionalization. The interaction between different probe ratios and the nanopore surface leads to changes in wettability, which in turn influences the fluid transport properties of the nanopore membranes. Data in the bar plot is presented as mean ± standard deviation values derived from results of three independent measurements (N=3). The error bars represent standard deviation values.

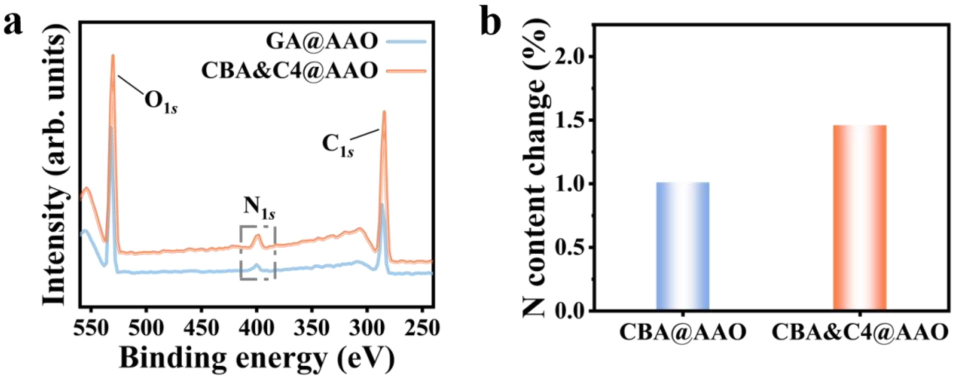

Supplementary Figure 2. XPS of CBA@AAO and CBA&C4@AAO. a XPS of the surfaces of CBA&C4@AAO (DNA mixing proportion 1:1) nanochannel membrane before and after preparation. **b** Changes of surface nitrogen content of CBA@AAO and CBA&C4@AAO N_CB_S before and after preparation.

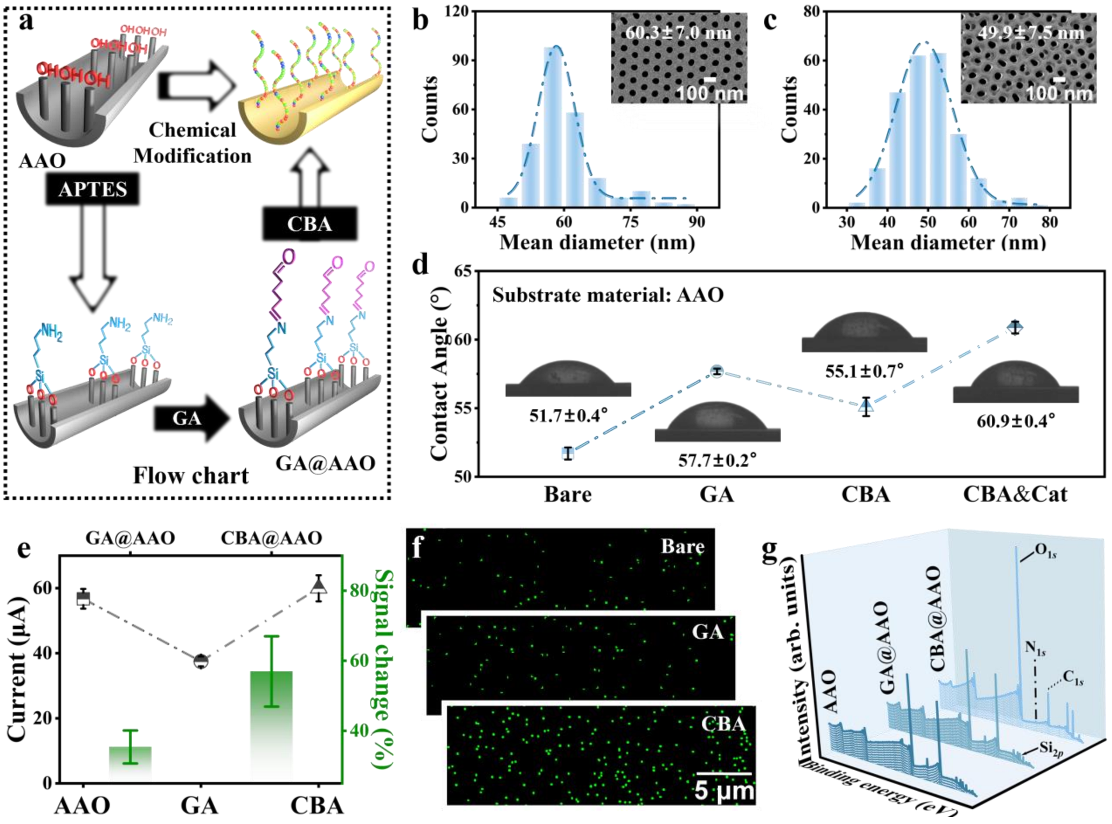

Supplementary Figure 3. Fabrication of the solid-state N_CB_S functionalized with DNA probe. **a** Schematic illustration of the preparation process for the CBA@AAO. SEM images and corresponding pore diameter distribution of the AAO nanochannel membrane before **b** and after **c** CBA functionalization. The complete SEM image is shown in Supplementary Figure 6. **d** Contact angles (CA) of the AAO membranes at each subsequent experimental step, with data obtained from three independent measurements. Insets show corresponding photographs of water droplet shapes on the membranes. **e** TmIC values at +2 V during *I*-*V* characteristic tests and changes in current signals before and after modification. EDS mapping (N_{1s}) **f** and XPS spectra **g** of the AAO, GA@AAO, and CBA@AAO membranes. The complete image of the EDS mapping is shown in Supplementary Figure 7. Data in the dot plots (d, e) and the bar plot (e) are presented as mean ± standard deviation values derived from results of three independent measurements (N=3). The error bars represent standard deviation values.

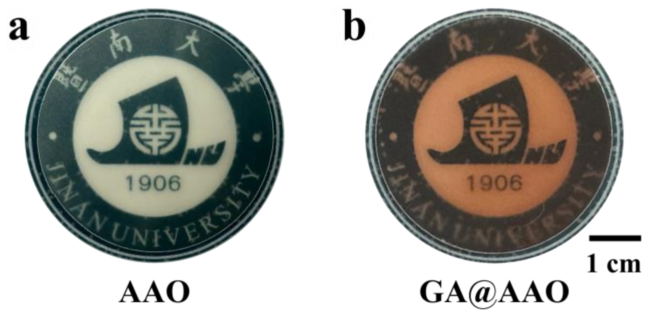

Supplementary Figure 4. AAO and GA@AAO nanochannel membrane diagram. The color change of anodized AAO nanochannel membrane **a** before and **b** after the modification of -CHO.

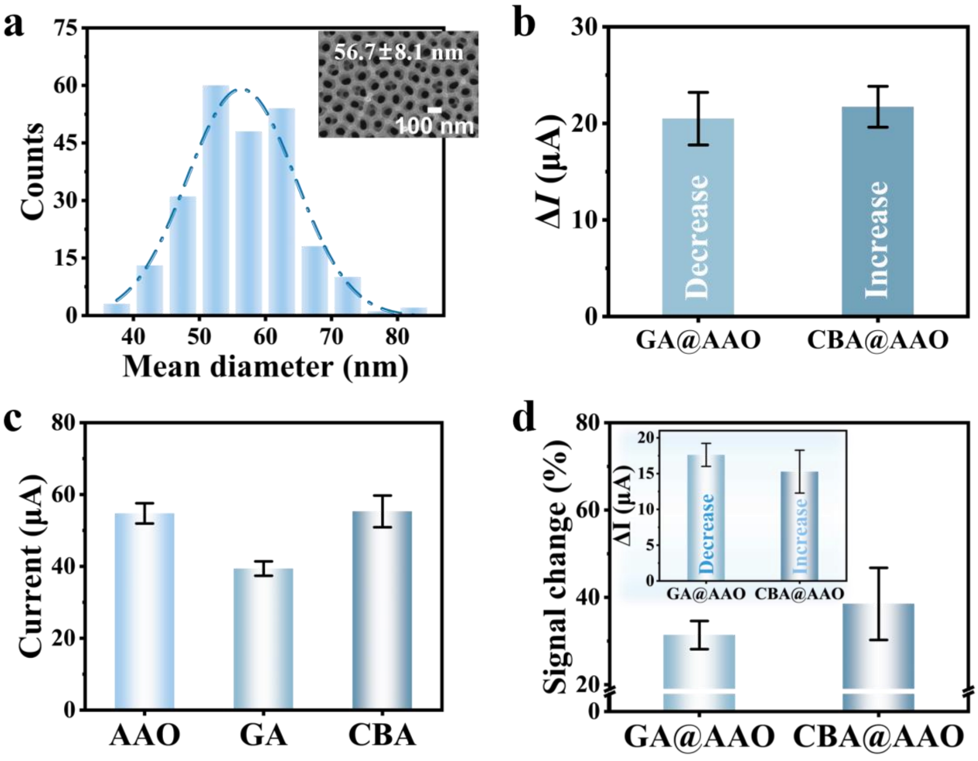

Supplementary Figure 5. Morphological and TmIC of different nanochannels. a SEM images and corresponding pore size distribution statistics of -CHO modified nanochannels (GA@AAO). **b** Changes in TmIC magnitude (based on *I-V* characteristics at +2 V) before and after the preparation of GA@AAO and CBA@AAO. **c** Steady-state TmIC passing through nanochannels at three stages (AAO, GA@AAO, and CBA@AAO) within 60 s under an applied voltage of +2 V. **d** Rate of change in TmIC signals before and after preparation of GA@AAO and CBA@AAO based on *I-T* characteristics. Insets illustrate changes in TmIC magnitude corresponding to the two stages. Data in the bar plots (b, c, d) are presented as mean ± standard deviation values derived from results of three independent measurements (N=3). The error bars represent standard deviation values.

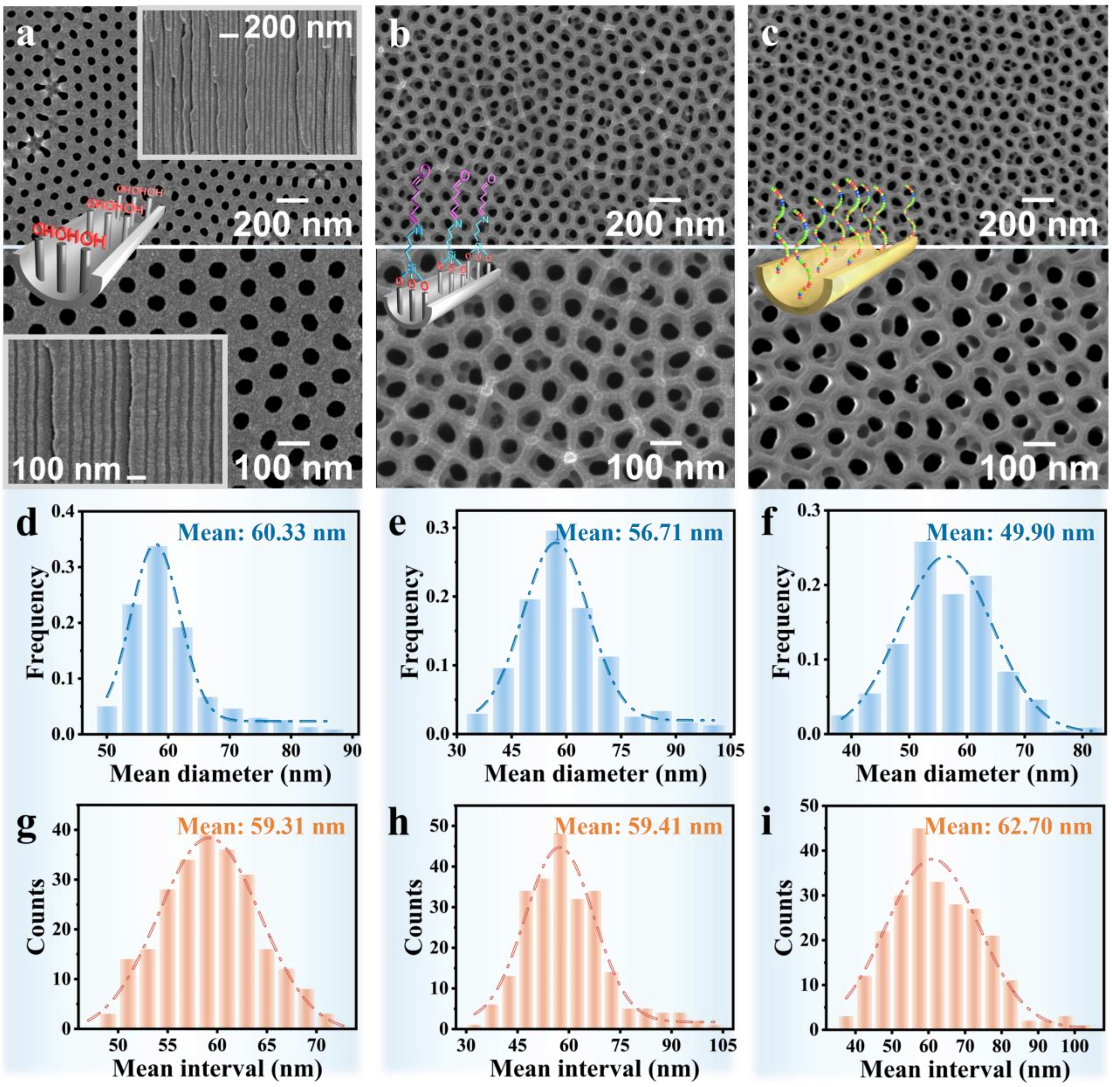

Supplementary Figure 6. SEM characterization and pore distribution analysis of nanochannels. a SEM images of the surface and cross-section of bare AAO membranes. SEM images of the surfaces of **b** GA@AAO and **c** CBA@AAO, respectively. Pore size distribution statistics for the three membrane states: **d** AAO, **e** GA@AAO, and **f** CBA@AAO, respectively. And pore spacing distribution statistics between adjacent pores in nanochannels of **g** AAO, **h** GA@AAO, and **i** CBA@AAO.

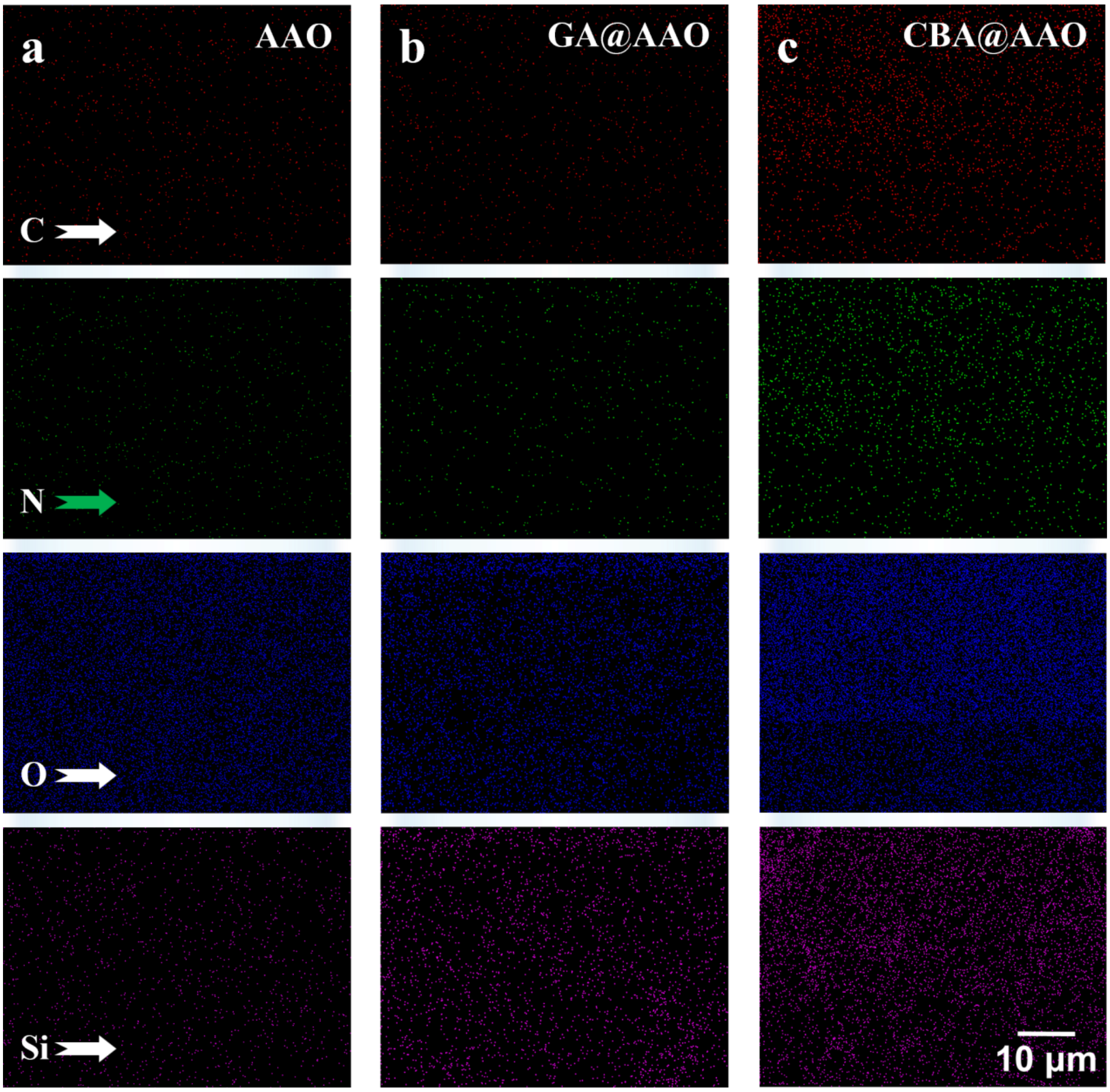

Supplementary Figure 7. EDS mapping of nanochannel membranes. EDS mapping images of the surface of nanochannel films in **a** AAO, **b** GA@AAO and **c** CBA@AAO states, respectively. Qualitative analysis of C, N, O and Si elements on the membrane surface.

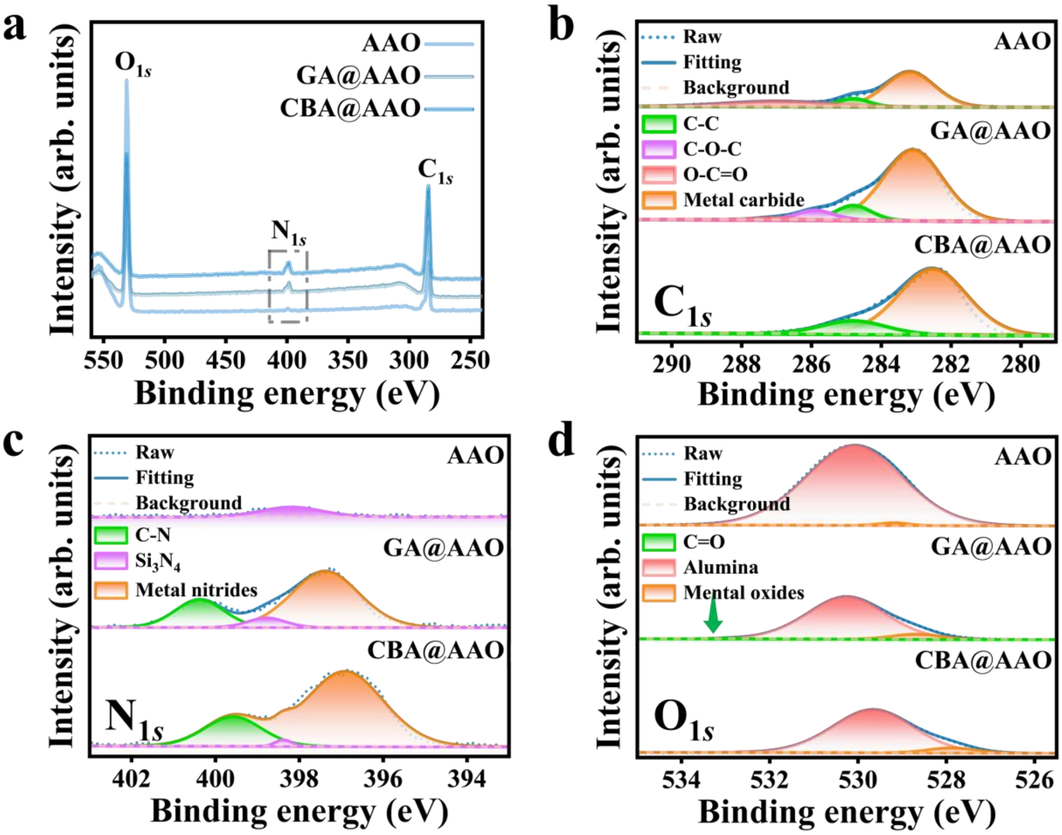

Supplementary Figure 8. XPS of the surfaces of nanochannel membranes. a Full XPS spectra of the surface of the nanochannel films in three states before and after functionalization. Narrow XPS spectrum near **b** C_{1s}, **c** N_{1s} and **d** O_{1s} peaks on the surface of each nanochannel membrane. The XPS results showed that CBA was successfully functionalized on AAO membrane, and CBA@AAO N_CB_S was prepared.

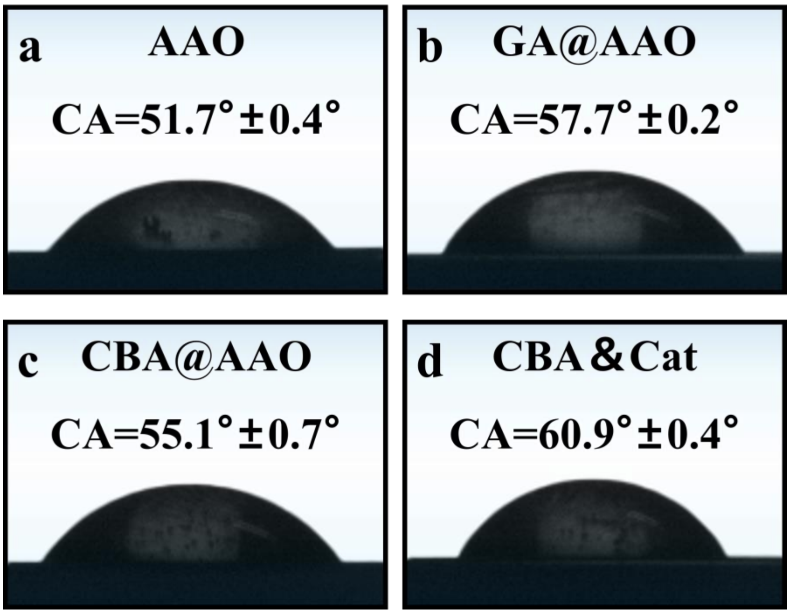

Supplementary Figure 9. CA test diagram. a AAO; **b** GA@AAO; **c** CBA@AAO; **d** CBA@AAO interaction with cathinone.

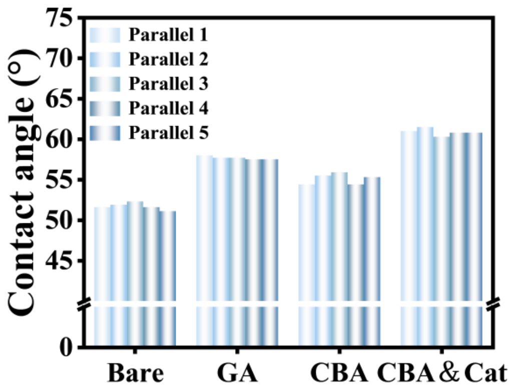

Supplementary Figure 10. Parallel experiments of five groups of CA test. Before and after functionalization of N_CB_S and after interaction with cathinone.

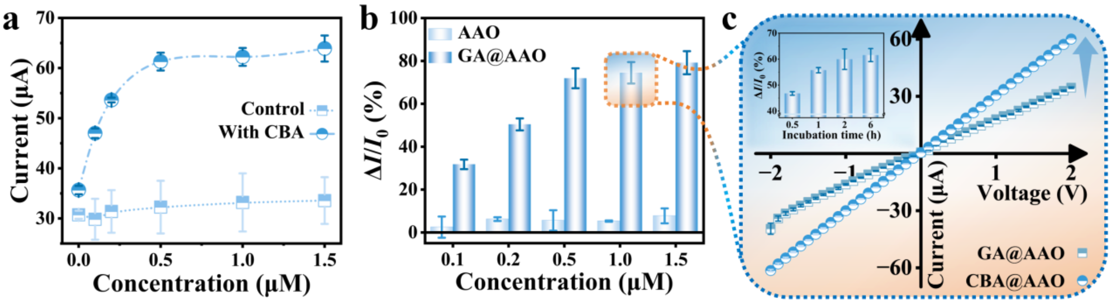

Supplementary Figure 11. TmIC changes of GA@AAO and AAO upon CBA incubation. a Current changes of rapid response *I*-*V* (+2 V) after 2 h incubation of GA@AAO in Tris-HCl (10 mM pH 7.4, 0.5 M NaCl, 1 mM $MgCl_2$) solutions with different concentrations of CBA. The control group was incubated in Tris-HCl without CBA to exclude environmental factors and solvent effects. **b** Rate of current change of rapid response *I*-*V* (+2 V) after 2 h incubation of AAO and GA@AAO in Tris-HCl solutions containing different concentrations of CBA. Non-specific adsorption of CBA was excluded. **c** *I*-*V* characteristics of CBA@AAO prepared with GA@AAO and 1 μM CBA. The inset shows the time-dependent incubation of GA@AAO and 1 μM CBA. Data in the dot plot (a, c) and the bar plots (b, c) are presented as mean ± standard deviation values derived from results of three independent measurements (N=3). The error bars represent standard deviation values.

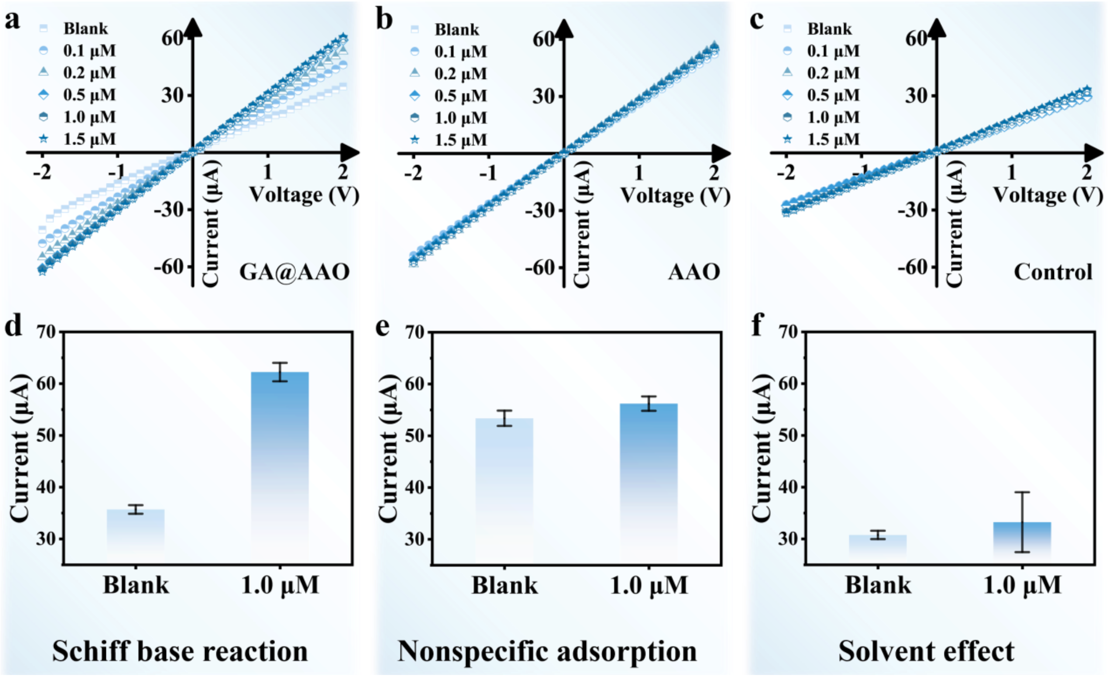

Supplementary Figure 12. Specific fabrication of CBA@AAO. **a** *I-V* characteristics of GA@AAO incubated with different concentrations (0.1, 0.2, 0.5, 1.0 and 1.5 μM) of CBA for 30 min. **b** *I-V* characteristics of AAO incubated with different concentrations of CBA for 30 min. **c** GA@AAO *I-V* characteristics after incubation for 30 min without different concentrations of CBA. **d** Current signal before and after incubation of GA@AAO with 1.0 μM CBA. **e** Current signal before and after incubation of AAO with 1.0 μM CBA. **f** GA@AAO current signals before and after soaking in 10 mM Tris-HCl (pH 7.4) solution for 2 h. Data in the bar plots (d, e, f) are presented as mean ± standard deviation values derived from results of three independent measurements (N=3). The error bars represent standard deviation values.

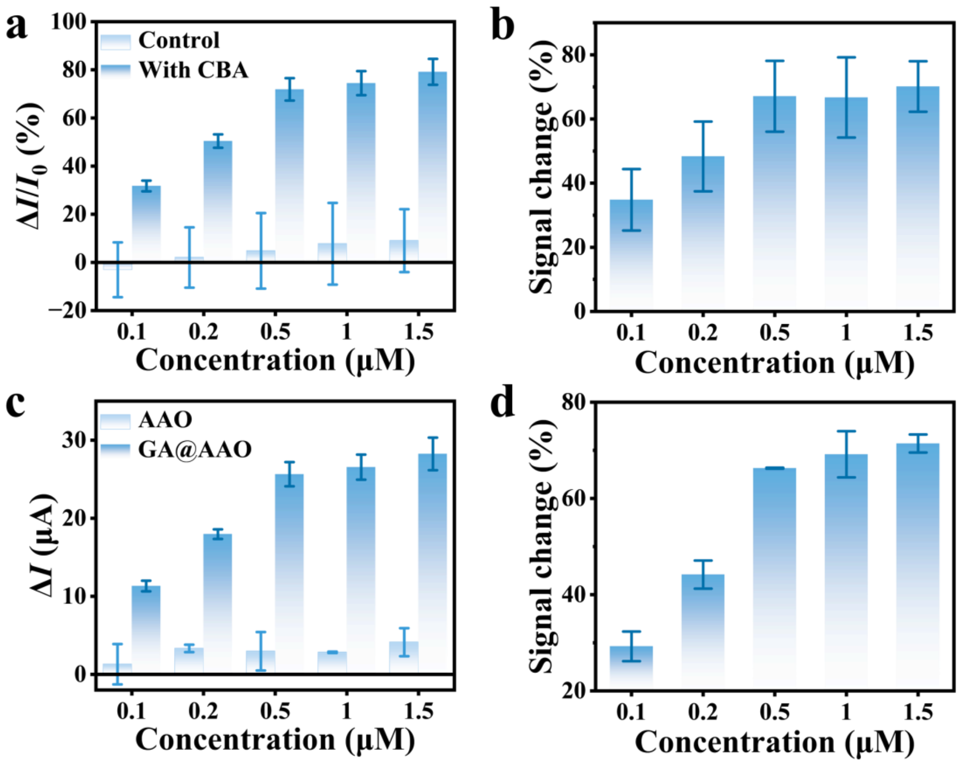

Supplementary Figure 13. TmIC changes of AAO and GA@AAO with various CBA concentrations. **a** Changes in the current signal ($\Delta I/I_0$) after incubating GA@AAO with different concentrations (0.1, 0.2, 0.5, 1.0, and 1.5 μM) of CBA. The control group involves incubating GA@AAO in Tris-HCl (0.01 M, pH 7.4) without CBA for the same duration as the CBA treatments to eliminate environmental influences, including the solvent (Tris-HCl) interference with the current signal of CBA@AAO. **b** Changes in ionic current signal ($\Delta I/I_0$) of CBA@AAO prepared by incubating GA@AAO with different concentrations of CBA, after subtracting the interference current signal caused by environmental influences. **c** Net increase in current changes after incubating AAO and GA@AAO with different concentrations of CBA, eliminating the non-specific adsorption of CBA on the nanochannel membrane, which instead is covalently grafted into the nanochannels through Schiff base reactions. **d** Ionic current signal of CBA@AAO prepared by incubating GA@AAO with CBA (covalently grafted through Schiff base reactions) after subtracting the signal changes caused by the interaction of AAO with different concentrations of CBA (non-specific adsorption). Data in the all bar plot are presented as mean ± standard deviation values derived from results of three independent measurements (N=3). The error bars represent standard deviation values.

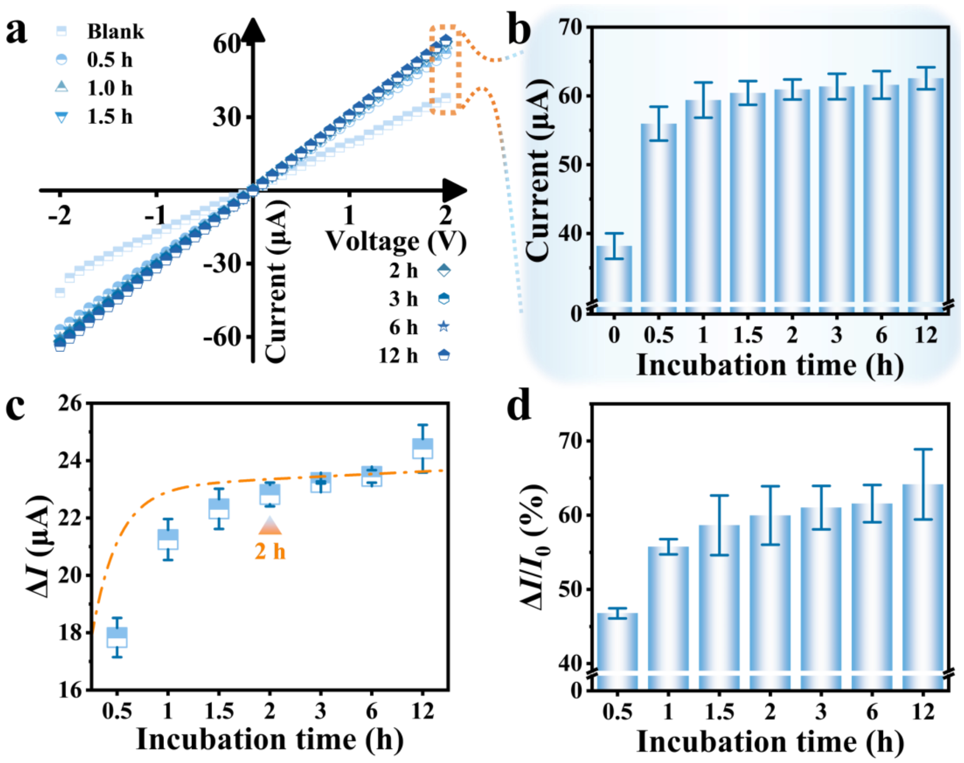

Supplementary Figure 14. Optimization of CBA incubation time. **a** *I*-*V* curves of GA@AAO incubated with 1 μM CBA for different times (0.5, 1.0, 1.5, 2, 3, 6, and 12 h). **b** TmIC at +2 V through CBA@AAO functionalized for different times based on the *I*-*V* characteristics. **c** Current changes (ΔI) at +2 V before and after functionalizing CBA@AAO for different times. The optimal incubation time is shown to be 2 h. **d** Changes in ionic current signal ($\Delta I/I_0$) of CBA@AAO prepared with different incubation times. Data in the dot plot (c) and the bar plots (b, d) are presented as mean ± standard deviation values derived from results of three independent measurements (N=3). The error bars represent standard deviation values.

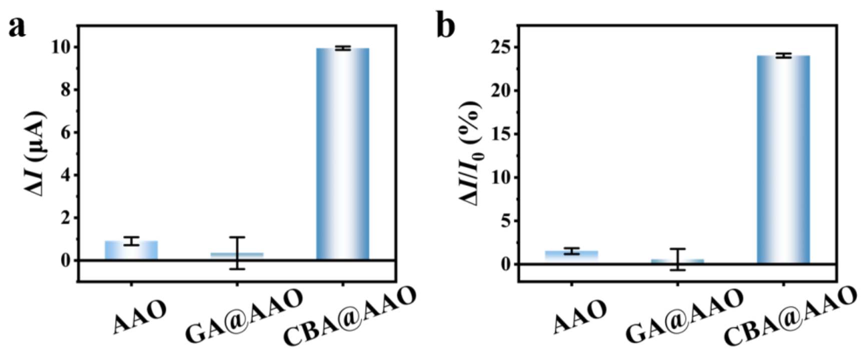

Supplementary Figure 15. Comparison of current magnitude and ionic current signals for $\mathbf{N_CB_S}$. AAO, GA@AAO and CBA@AAO investigated the changes of current magnitude **a** and ionic current signal **b** before and after the identification of 1 nM cathinone, respectively. Data in the bar plots are presented as mean ± standard deviation values derived from results of three independent measurements (N=3). The error bars represent standard deviation values.

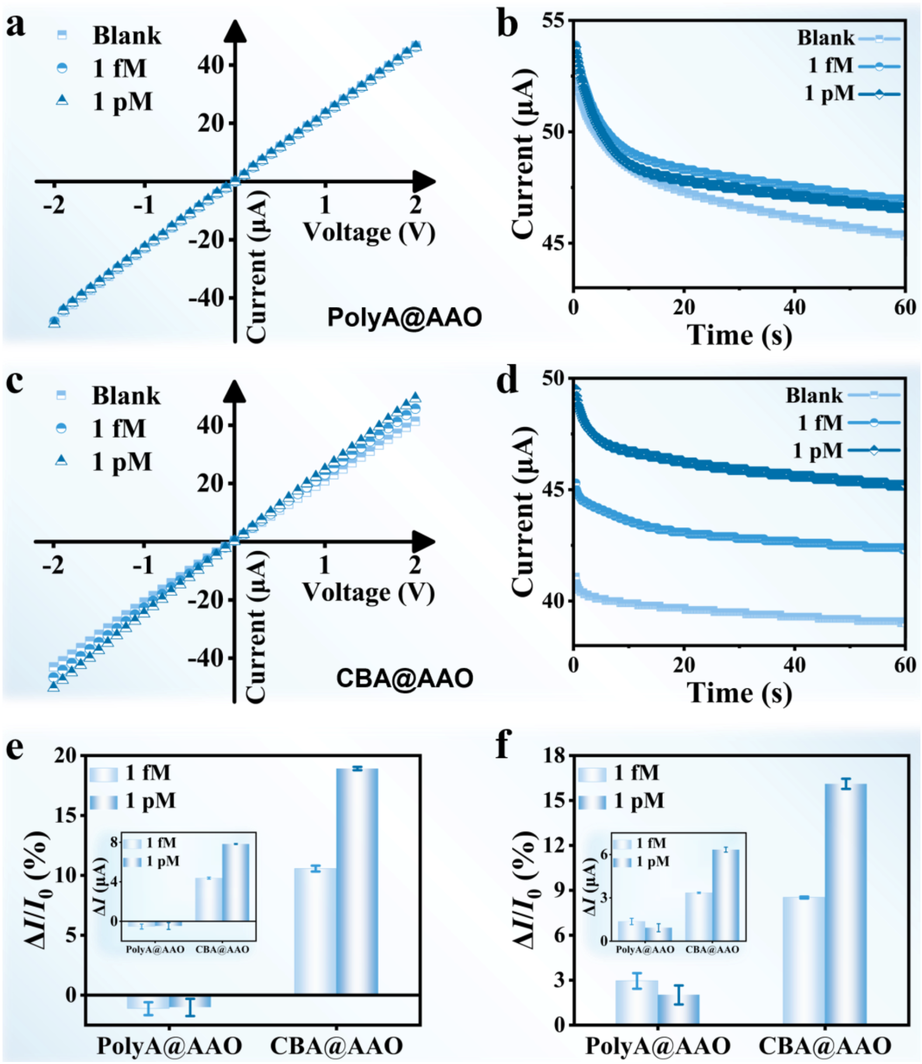

Supplementary Figure 16. PolyA@AAO and CBA@AAO N$_C$B$_S$ sensitivity. a PolyA@AAO and **c** CBA@AAO *I-V* characteristics before and after interaction with 1 fM and 1 pM cathinone, respectively. *I-T* characteristics of **b** PolyA@AAO and **d** CBA@AAO before and after interaction with 1 fM and 1 pM cathinone, respectively, with a period of 60 s. **e** and **f** are the changes of ionic current signals ($\Delta I/I_0$) before and after the recognition of two different concentrations of cathinone by the two sensors under the applied voltage +2 V in the *I-V* and *I-T* characteristics, respectively. The illustrations show the change of current magnitude (ΔI) before and after the action of the target object. Data in the bar plots (e, f) are presented as mean ± standard deviation values derived from results of three independent measurements (N=3). The error bars represent standard deviation values.

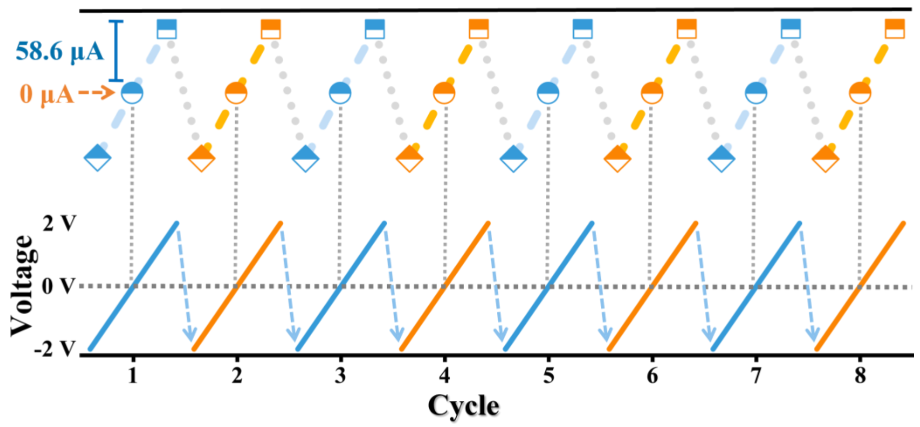

Supplementary Figure 17. Cycling of CBA@AAO for 1 pM cathinone direct detection.

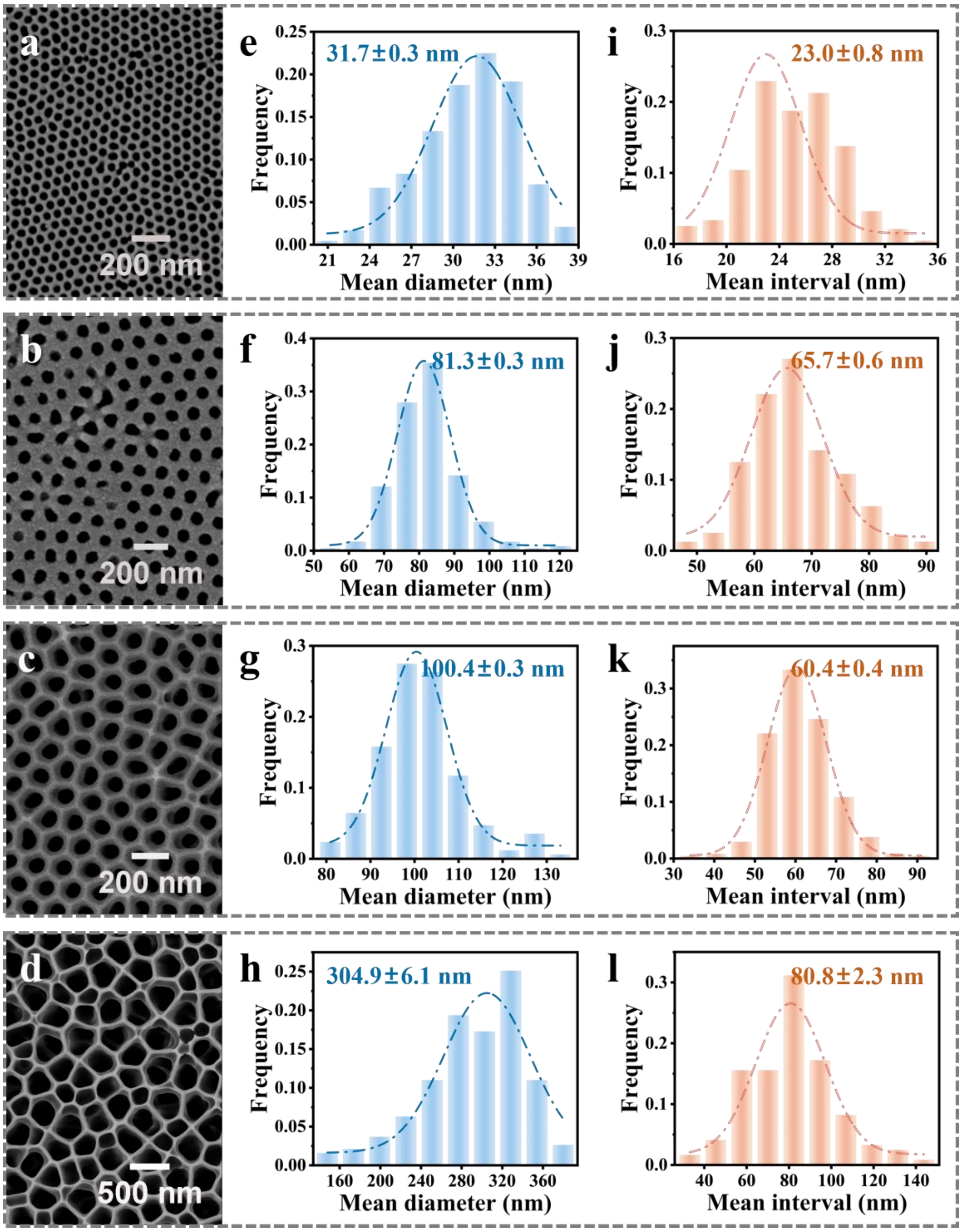

Supplementary Figure 18. Four different sizes of AAO nanochannel membranes. a AAO with aperture distribution of 20-30 nm. **e** and **i** are the corresponding pore distribution and pore gap size statistics of adjacent holes, respectively. **b** AAO with aperture distribution of 80-100 nm. **f** and **j** are the corresponding pore distribution and pore gap size statistics of adjacent holes respectively. **c** AAO with aperture distribution of 110-150 nm. **g** and **k** are the corresponding pore distribution and pore gap size statistics of adjacent holes, respectively. **d** AAO with an aperture distribution of 200-300 nm. **h** and **l** are the corresponding pore distribution and pore gap size statistics of adjacent holes, respectively.

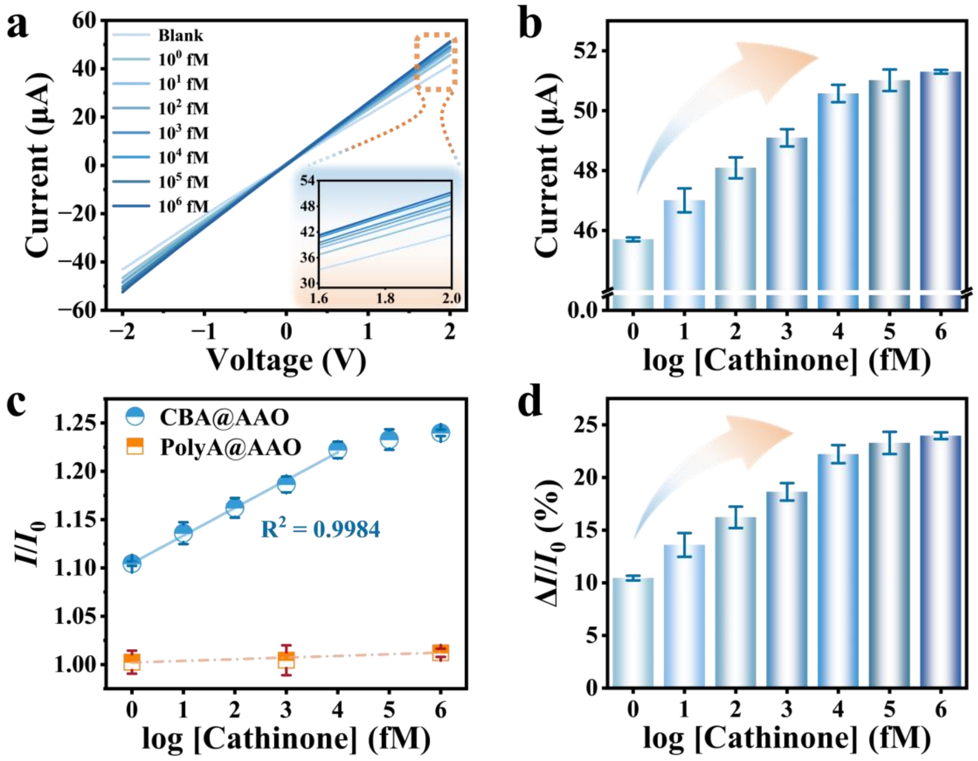

Supplementary Figure 19. TmIC response of CBA@AAO with different cathinone concentrations. a *I*-*V* characteristics of CBA@AAO in response to different concentrations of cathinone. The illustration is a partial enlargement of the current size at an applied voltage of 1.6 ~ 2.0 V. **b** Ionic current at +2 V at the *I*-*V* characteristic of CBA@AAO sensor in response to different concentrations of catinone. **c** The ionic current ratio (I/I_0) before and after the sensor recognizes the target at different concentrations, and the linear response range to the target. Compared with PolyA@AAO, CBA@AAO has remarkable sensitivity. **d** TmIC signal changes ($\Delta I/I_0$) of CBA@AAO after recognizing different concentrations of cathinone. Data in the dot plot (c) and the bar plot (b, d) are presented as mean ± standard deviation values derived from results of three independent measurements (N=3). The error bars represent standard deviation values.

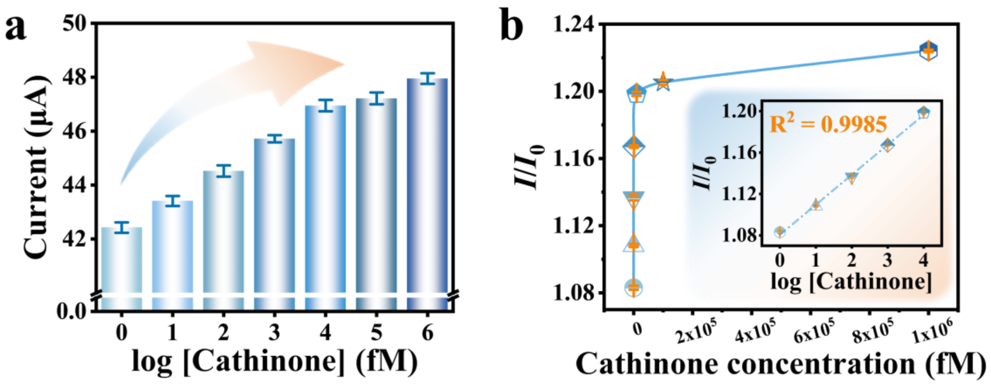

Supplementary Figure 20. *I-T* measurements of CBA@AAO with cathinone at +2 V. a *I-T* current recordings at an applied voltage of +2 V over a period of 60 s. Ionic current response of the CBA@AAO sensor to different concentrations of cathinone. **b** Current ratio (I/I_0) recorded at +2 V for different target concentrations of cathinone based on *I-T* characteristics. The inset shows the linear response of CBA@AAO to cathinone concentrations ranging from 1 to 10^4 fM, with R^2 = 0.9985. Data in the bar plot (a) and the dot plot (b) are presented as mean ± standard deviation values derived from results of three independent measurements (N=3). The error bars represent standard deviation values.

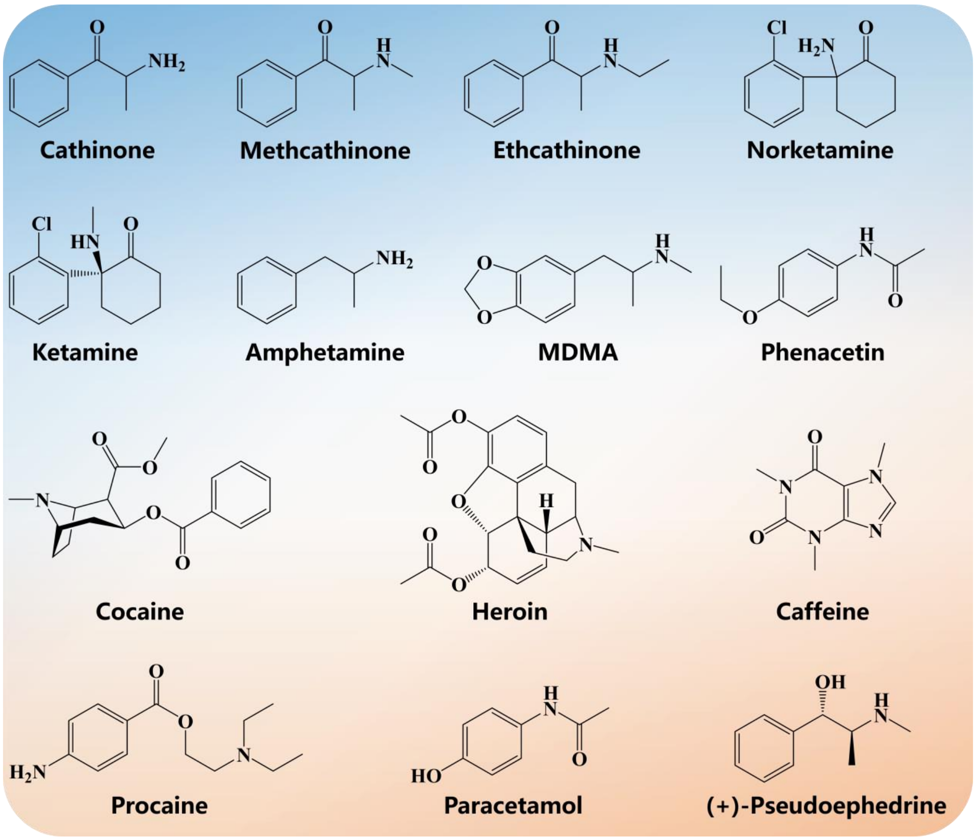

Supplementary Figure 21. Structures of the target analyte and 13 psychoactive drugs. Cathinone: Cat, Methcathinone: Met, Ethcathinone: Eth, Norketamine: Nor, Ketamine: Ket, Amphetamine: Amp, 3,4-Methylenedioxymethamphetamine: MDMA, Phenacetin: Phe, Cocaine: Coc, Heroin: Her, Caffeine: Caf, Procaine: Pro, Paracetamol: Par, (+)-Pseudoephedrine: PSE.

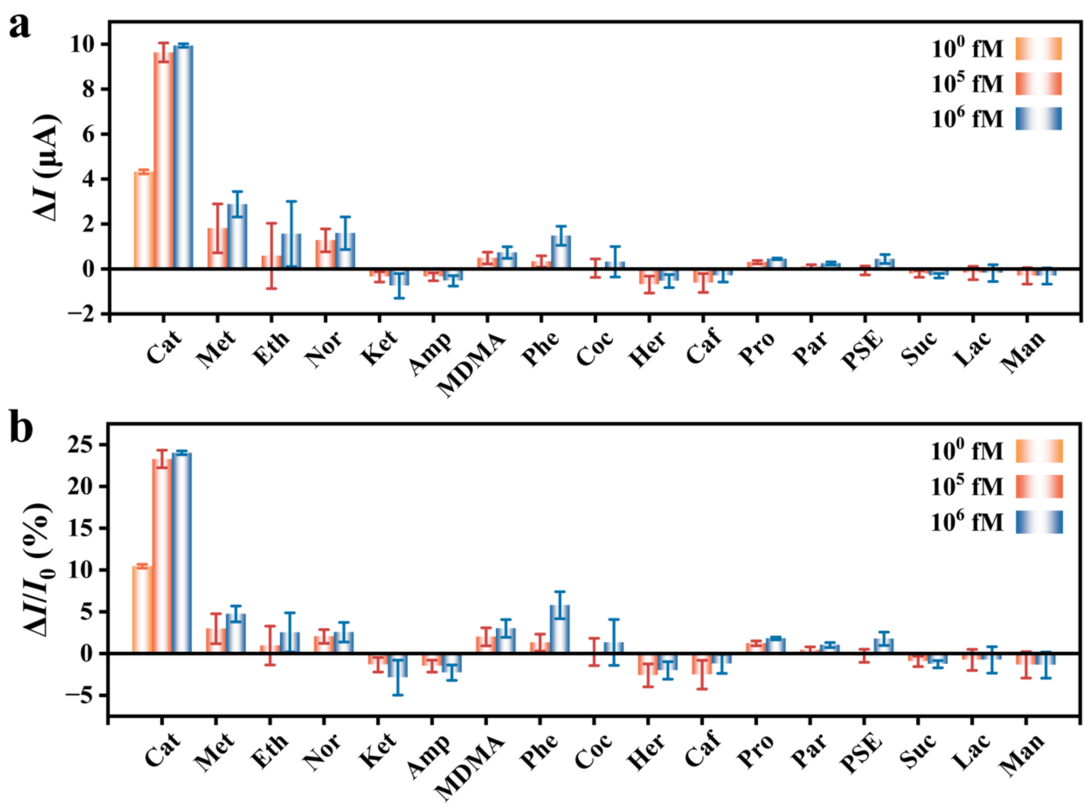

Supplementary Figure 22. Selectivity of the CBA@AAO. Comparison of 13 psychoactive drugs and 3 interfering substances, based on changes in current magnitude before and after recognition at +2 V from **a** *I*-*V* characteristics and **b** changes in ionic current signals. The concentration of cathinone was 1, 10^5, and 10^6 fM, while the concentrations of other interfering substances were 10^5 and 10^6 fM. Data in the bar plots are presented as mean ± standard deviation values derived from results of three independent measurements (N=3). The error bars represent standard deviation values.

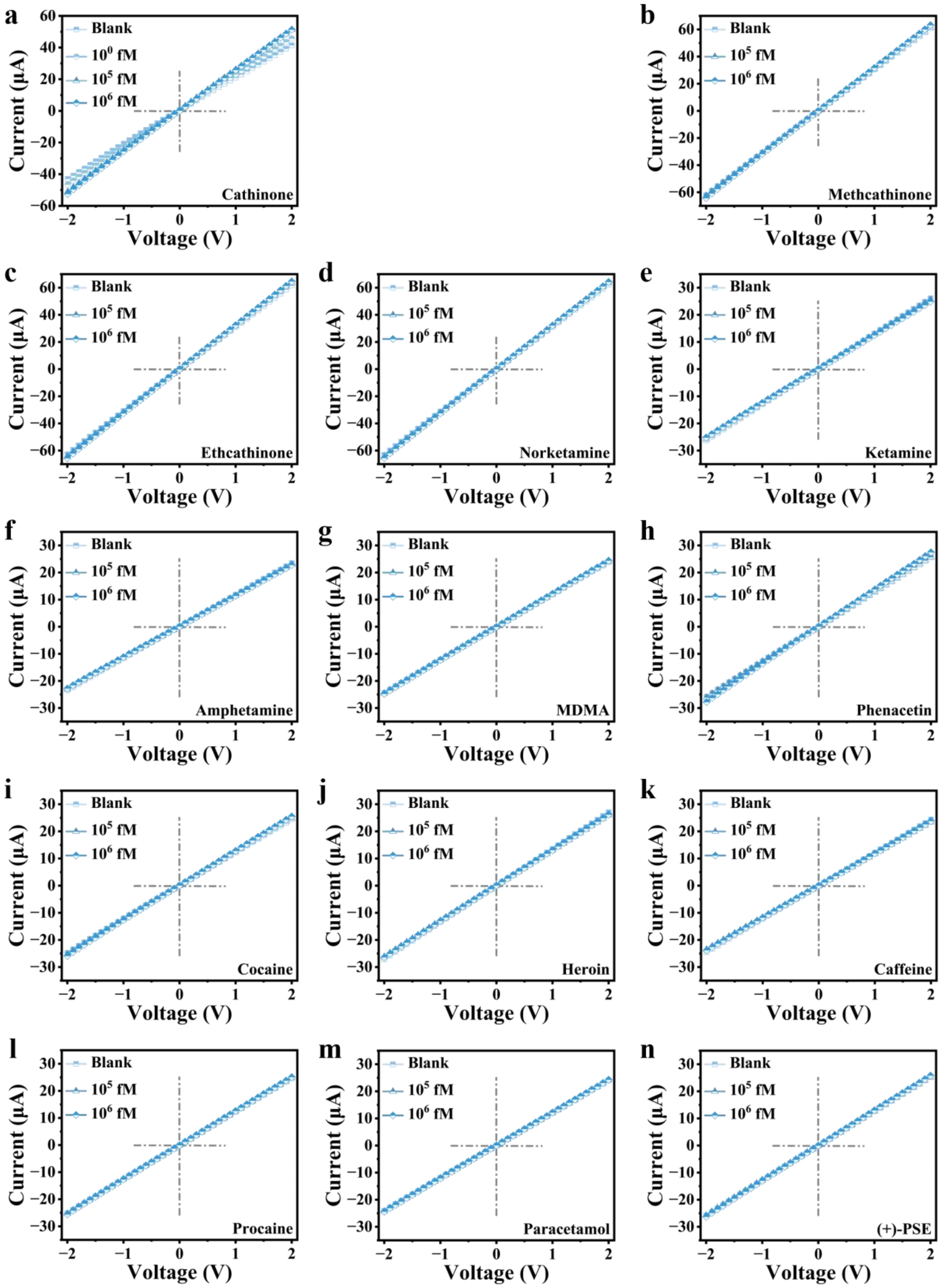

Supplementary Figure 23. ***I-V*** **of CBA@AAO with target and 13 psychoactive drugs.**

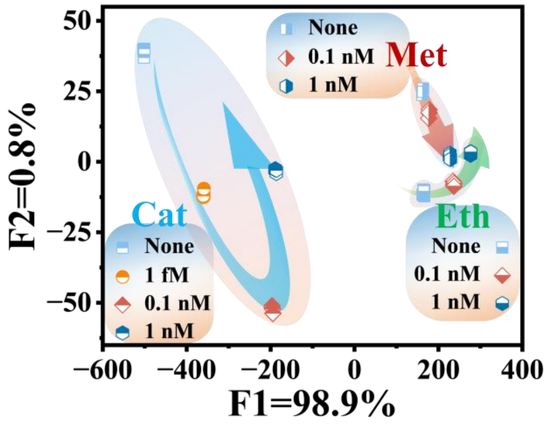

Supplementary Figure 24. Principal component analysis (PCA) score plot for distinguishing different concentrations of Cat, Met, and Eth. The clusters are dispersed along the direction of increasing concentration indicated by arrows, with an accuracy of 94%. Data were obtained from 5 separate parallel experiments (N=5).

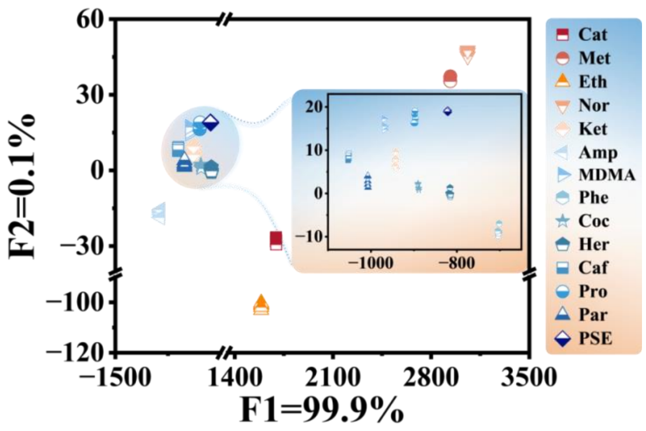

Supplementary Figure 25. PCA score plot was utilized to differentiate 14 different drug analytes. All drugs have a concentration of 1 nM and the classification accuracy of 100%. Data were obtained from 5 separate parallel experiments (N=5).

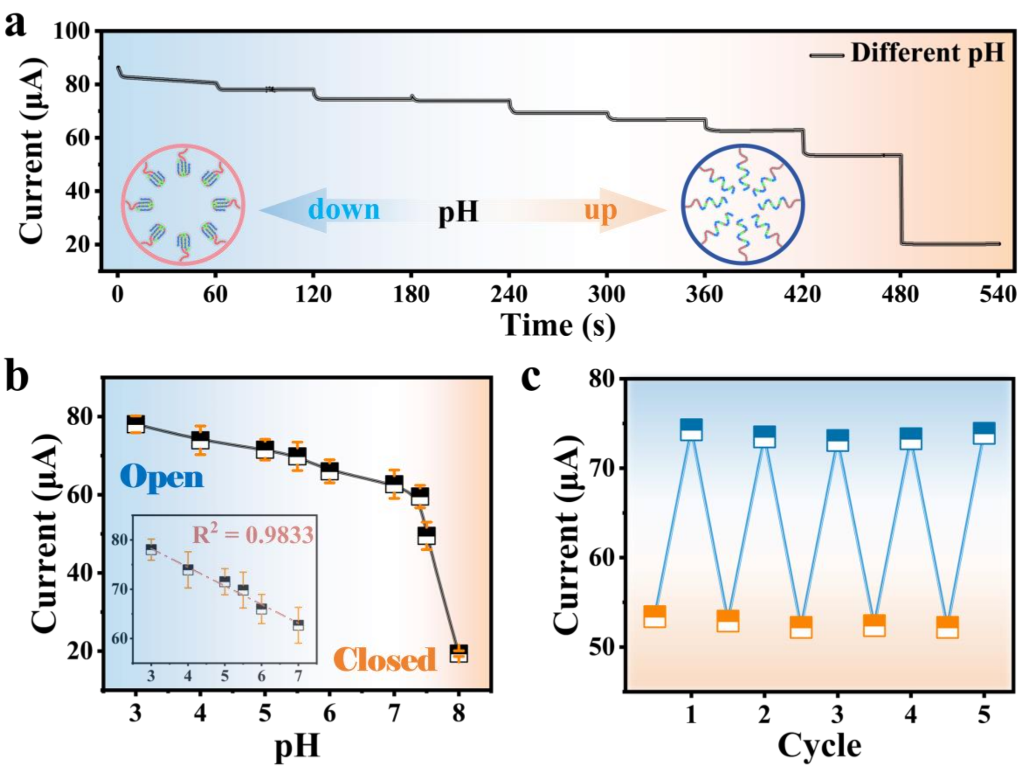

Supplementary Figure 26. pH-responsive C4@AAO. a *I-T* response of C4@AAO to different pH values (3, 4, 5, 5.5, 6, 7, 7.4, 7.5 and 8) under an applied voltage of +2 V, with each pH response cycle lasting for 60 s. Inset shows the reversible change in effective pore diameter of C4@AAO with pH variation. **b** TmIC magnitude of C4@AAO sensor in response to different pH values under +2 V. Inset displays the linear fitting of the sensor within the pH range of 3 ~ 7, with R^2 = 0.9833. Directly correlates to the opening of the functionalized nanochannel, leading to an increase in effective pore diameter at low pH values. As pH increases, the C4@AAO nanochannel gradually closes, resulting in a decrease in ionic current. **c** Cycling stability of C4@AAO under two pH states (pH 5.5 and 7.5), based on *I-T* characteristics under an applied voltage of +2 V. Data in the dot plot (b) is presented as mean ± standard deviation values derived from results of three independent measurements (N=3). The error bars represent standard deviation values.

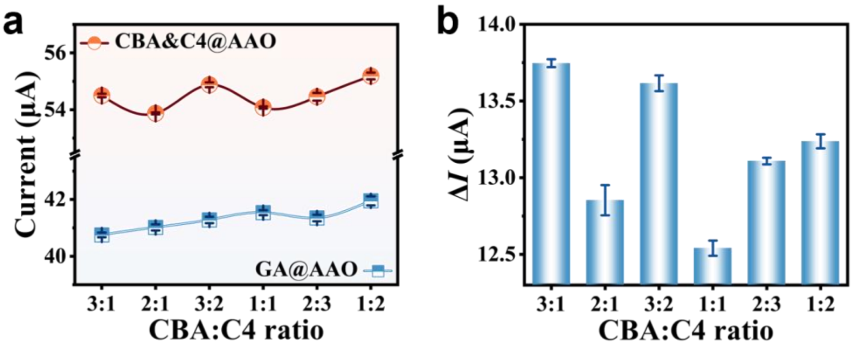

Supplementary Figure 27. TmIC changes of N_CB_S with varying CBA:C4 DNA ratios. a The ionic current of DNA molecules with different proportions at +2 V before and after functionalization. **b** Current changes before and after functionalization. Data in the dot plot (a) and the bar plot (b) are presented as mean ± standard deviation values derived from results of three independent measurements (N=3). The error bars represent standard deviation values.

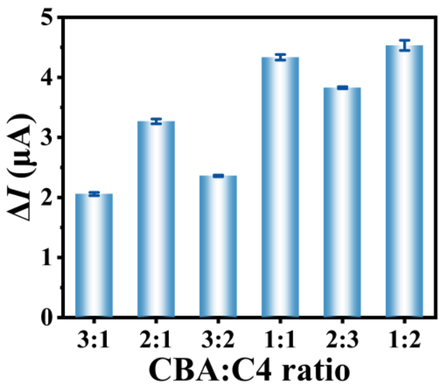

Supplementary Figure 28. Current changes of N_CB_S modified with different proportions of DNA molecules before and after response to 1 pM cathinone. Data in the bar plot is presented as mean ± standard deviation values derived from results of three independent measurements (N=3). The error bars represent standard deviation values.

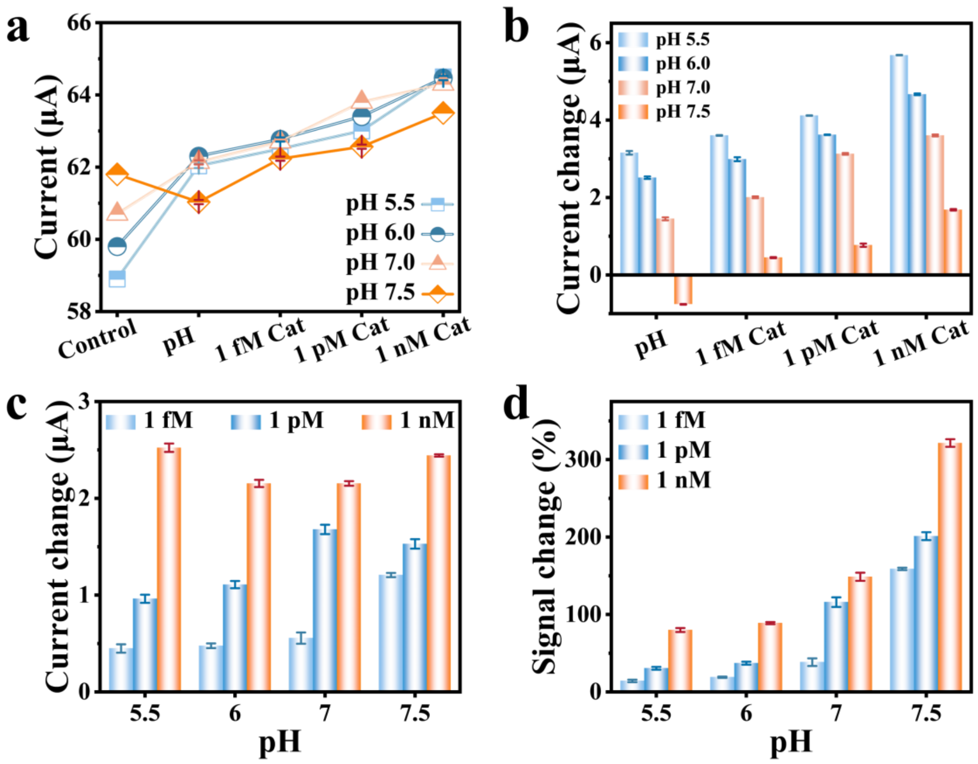

Supplementary Figure 29. TmIC responses of CBA&C4@AAO and its dependence on pH and target concentration. a Based on the *I-V* characteristics, transmembrane ionic current response of CBA&C4@AAO sensor under an applied voltage of +2 V for four different conditions. The control group is Tris-HCl (0.01 M) at pH 7.4. **b** Current changes of the sensor after recognition of target substances under four different conditions. **c** Current changes of the sensor after cathinone recognition at different concentrations (1 fM, 1 pM, and 1 nM) under four different pH values. **d** Signal changes of CBA&C4@AAO sensor after cathinone recognition at different concentrations under different pH conditions. Data in the dot plot (a) and the bar plots (b, c, d) are presented as mean ± standard deviation values derived from results of three independent measurements (N=3). The error bars represent standard deviation values.

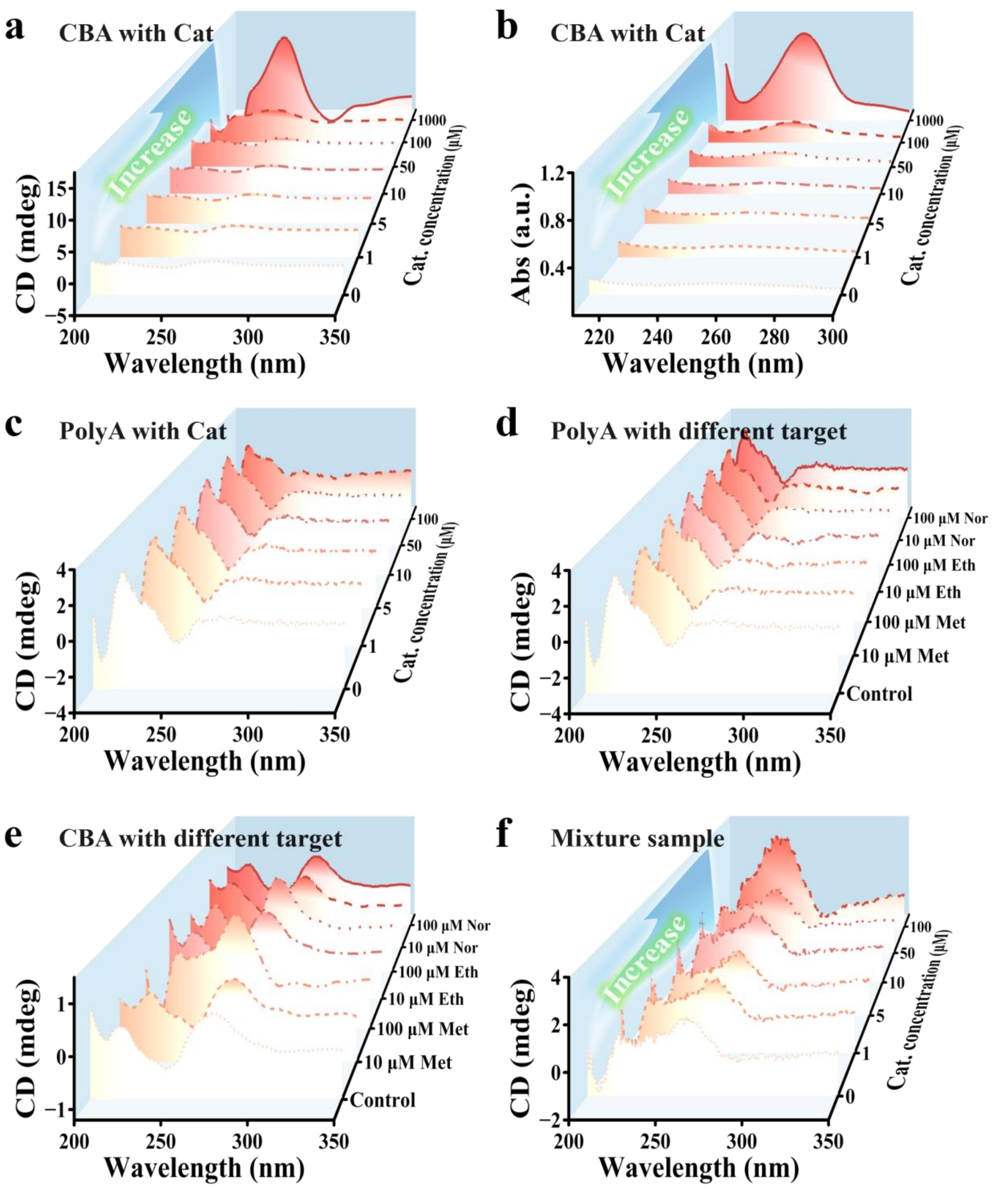

Supplementary Figure 30. CD and UV spectral responses of CBA and PolyA upon interaction with cathinone (Cat) and its analogs. a Changes in CD spectral signals of CBA after interaction with different concentrations of cathinone. **b** UV absorption changes of CBA after binding to different concentrations of cathinone. **c** CD signal changes of PolyA after interaction with different concentrations of cathinone. **d** CD signal changes of PolyA after interaction with 10 and 100 µM of methcathinone (Met), ethcathinone (Eth), and norketamine (Nor). **e** CD signal changes of CBA after interaction with 10 and 100 µM of methcathinone, ethcathinone, and norketamine. **f** CD response signals of CBA to different concentrations of target cathinone in a mixed sample, where the mixed sample (blank group) consists of 1 µM CBA and 100 µM of methcathinone, ethcathinone, and norketamine.

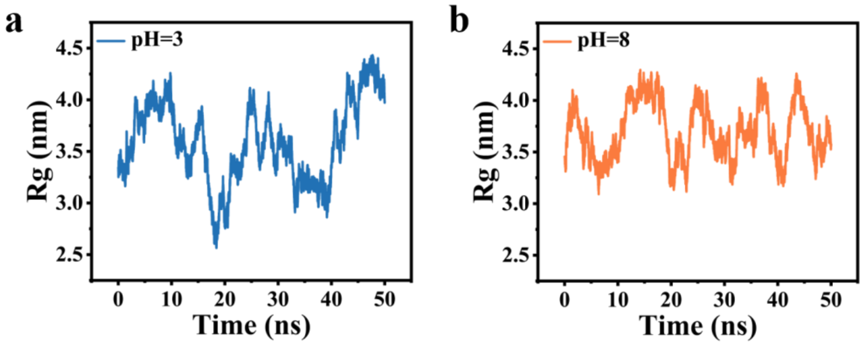

Supplementary Figure 31. Molecular dynamics cyclotron radius diagram of the complex. a pH=3; **b** pH=8. The radius of gyration (Rg) is used to assess the compactness of the structure. A larger Rg value indicates a looser structure, while a smaller value indicates a more tightly packed conformation. The figure shows that after equilibration, the complex exhibits minimal fluctuation, with the system remaining stable at approximately 3.64 nm (pH=3) and 3.69 nm (pH=8).

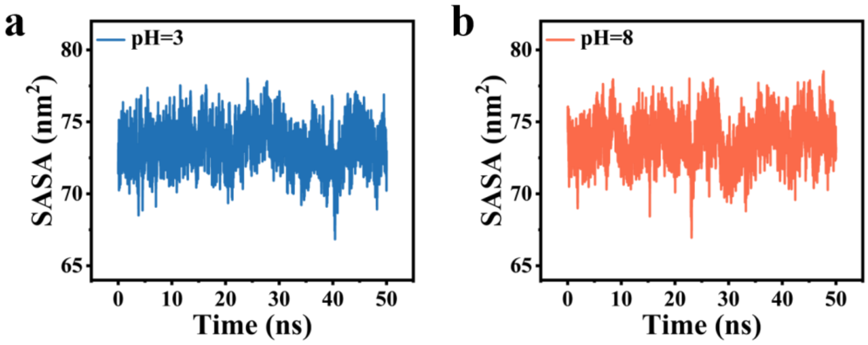

Supplementary Figure 32. Average solvent-accessible and total surface area of protein residues in the experimental system. a pH=3; **b** pH=8. Solvent-accessible surface area (SASA) refers to the area of the protein surface that is accessible to solvent molecules, serving as a measure of the protein's surface exposure to the solvent. A lower SASA value indicates a more compact structure, while a higher value suggests a more extended conformation. As shown in the figure, the solvent-accessible surface area of the entire system remains relatively stable, maintaining values of 73.35 nm^2 (pH=3) and 73.71 nm^2 (pH=8).

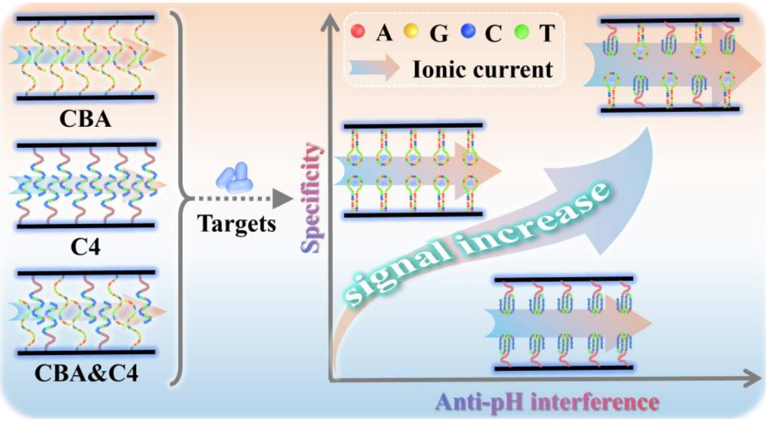

Supplementary Figure 33. Schematic diagram of co-calibration of cathinone and pH with dual-DNA probes N_CB_S.

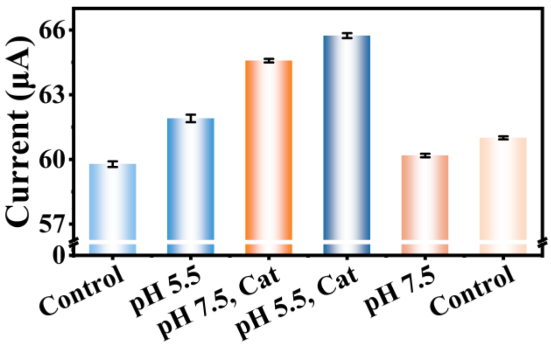

Supplementary Figure 34. TmIC variation of CBA&C4@AAO under different conditions. Data in the bar plot is presented as mean ± standard deviation values derived from results of three independent measurements (N=3). The error bars represent standard deviation values.

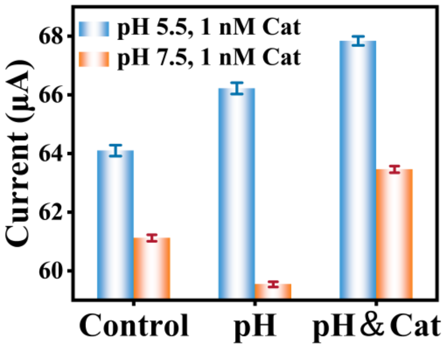

Supplementary Figure 35. Orthogonal responsiveness of CBA&C4@AAO. The conditions include pH 5.5, pH 7.5, pH 5.5 with 1 nM cathinone, and pH 7.5 with 1 nM cathinone. The control group is the transmembrane electrical signal in a test environment with pH 7.4. The N_CB_S was prepared based on asymmetric solution modification. Data in the bar plot is presented as mean ± standard deviation values derived from results of three independent measurements (N=3). The error bars represent standard deviation values.

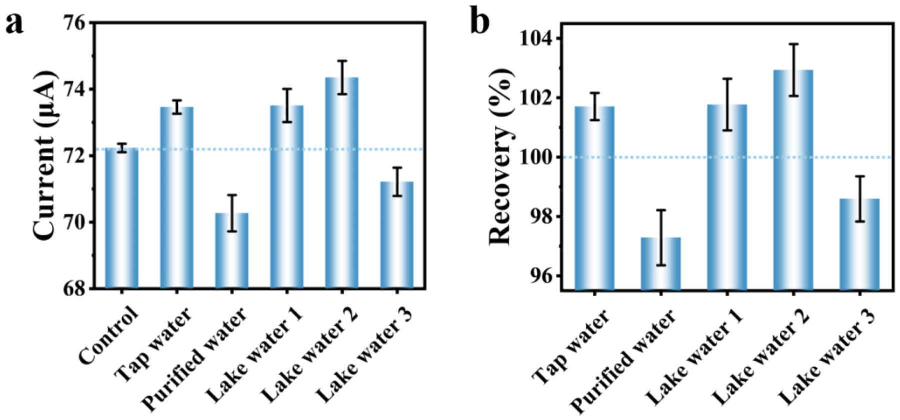

Supplementary Figure 36. Spike-and-recovery experience. a TmIC signal of CBA&C4@AAO N_CB_S responding to 1 pM cathinone in different water samples under +2V voltage. **b** Recovery rate of added standard in different water samples. The five water samples are tap water (pH=7.16), pure water (pH=6.97), and water samples from the upstream (pH=7.49), middle (pH=8.02) and downstream (pH=8.36) of Jinghu Lake on the campus of Jinan University. The single test runs parallel to three groups. Data in the bar plots are presented as mean ± standard deviation values derived from results of three independent measurements (N=3). The error bars represent standard deviation values.

3. Supplementary Tables

Supplementary Table 1. Sequences of all single-stranded DNA oligonucleotides. CBA, PolyA, and C4 DNA serve as the functionalizing probe DNAs for the N_CB_S, while CBA I, PolyA I, and C4 DNA I are the probe DNAs used for CD spectroscopy analysis.

Name	DNA Sequence
**CBA**<br>Cathinone binding aptamer	5'-NH_2-$(CH_2)_6$-ACT GAG AAG TGT GAT TCA GTA TGT TTT CCG AAG T-3'
**CBA I**<br>Cathinone binding aptamer I	5'-ACT GAG AAG TGT GAT TCA GTA TGT TTT CCG AAG T-3'
**PolyA**<br>Similar to CBA full base A	5'-NH_2-$(CH_2)_6$-AAA AAA AAA AAA AAA AAA AAA AAA AAA AAA AAA A-3'
**PolyA I**<br>Similar to CBA full base A I	5'-AAA AAA AAA AAA AAA AAA AAA AAA AAA AAA AAA A-3'
**C4 DNA**<br>pH responsiveness	5'-NH_2-$(CH_2)_6$-AAA AAA AAA ACC CTT ACC CTT ACC CTT ACC C-3'
**C4 DNA I**<br>pH responsiveness I	5'-AAA AAA AAA ACC CTT ACC CTT ACC CTT ACC C-3'

Supplementary Table 2. XPS date of the CBA&C4@AAO (CBA:C4 DNA = 1:1) before and after preparation.

Name	Start BE	Peak BE	End BE	Height CPS	FWHM eV	Area (P) CPS.eV	At. %
**CBA&C4@AAO before preparation**							
C_{1s}	297.98	283.71	279.18	37564.77	2.15	101790.92	52.33
N_{1s}	409.98	398.07	392.18	3237.82	2.31	11093.84	3.67
O_{1s}	544.98	530.53	525.18	72611.05	2.67	206928.75	44
**CBA&C4@AAO N_CB_S**							
C_{1s}	297.98	283.86	279.18	51809.96	2.28	138769.07	69.27
N_{1s}	409.98	398.08	392.18	4952.13	2.01	15957.73	5.13
O_{1s}	544.98	530.96	525.18	48219.55	2.26	123979.58	25.6

Supplementary Table 3. Pore diameter, density and porosity for nanochannels.

	Actual pore diameter (nm)	Mean pore diameter (nm)	Pore density (pores/cm^2)	Poriness (%)
AAO	48.1-88.3	60.3±7.0	8.8×10^{9}	17.5
GA@AAO	36.8-83.5	56.4±8.1	1.0×10^{10}	18.9
CBA@AAO	34.3-77.6	49.9±7.5	1.2×10^{10}	21.6

Supplementary Table 4. XPS date of the AAO.

Name	Start BE	Peak BE	End BE	Height CPS	FWHM eV	Area (P) CPS.eV	At. %
C_{1s}	294.08	283.40	277.08	127616.90	3.10	510649.06	25.42
N_{1s}	408.58	398.32	390.08	5846.15	3.43	32158.38	1.03
O_{1s}	536.08	530.16	523.08	677307.87	3.26	2378535.57	48.95
Al_{2p}	79.08	73.03	65.08	103110.6	2.79	313553.52	24.6

Supplementary Table 5. XPS date of the GA@AAO.

Name	Start BE	Peak BE	End BE	Height CPS	FWHM eV	Area (P) CPS.eV	At. %
C_{1s}	291.80	283.41	276.30	272310.03	3.19	974194.17	56.87
N_{1s}	409.30	398.02	389.30	24776.90	4.21	119385.47	4.49
O_{1s}	535.30	530.16	522.30	332384.34	3.03	1099010.59	26.53
Al_{2p}	77.80	72.78	62.30	23439.81	2.75	80614.35	7.42
Si_{2p}	107.30	100.23	92.30	24242.76	2.66	80815.28	4.7

Supplementary Table 6. XPS date of the CBA@AAO.

Name	Start BE	Peak BE	End BE	Height CPS	FWHM eV	Area (P) CPS.eV	At. %
C_{1s}	289.60	283.03	275.60	258702.37	3.39	960756.46	56.25
N_{1s}	407.60	397.36	391.60	34176.57	3.99	145850.64	5.50
O_{1s}	535.60	529.74	521.60	326336.15	3.16	1136821.12	27.52
Al_{2p}	78.10	72.06	62.10	20826.09	2.91	70055.77	6.46
Si_{2p}	107.10	99.87	91.60	22137.09	2.92	73290.88	4.27

Supplementary Table 7. XPS narrow spectra date of the AAO.

Name	Start BE	Peak BE	End BE	Height CPS	FWHM eV	Area (P) CPS.eV	At. %
Metal carbide	297.30	283.17	278.50	34286.23	1.68	62568.93	22.71
C-C	297.30	284.80	278.50	7978.49	1.04	8978.56	3.26
O-C=O	297.30	287.00	278.50	5236.68	3.50	19861.85	7.23
Si_3N_4	409.30	398.19	391.50	1388.27	1.74	2615.38	0.61
Al_2O_3	544.30	530.08	524.50	155890.40	2.58	435576.75	65.39
Mental oxides	544.30	529.18	524.50	5908.22	0.83	5294.4	0.79

Supplementary Table 8. XPS narrow spectra date of the GA@AAO.

Name	Start BE	Peak BE	End BE	Height CPS	FWHM eV	Area (P) CPS.eV	At. %
Metal carbide	297.20	283.08	278.40	69104.11	2.01	150168.07	53.71
C-C	297.20	284.80	278.40	14266.21	1.17	18135.54	6.49
C-O-C	297.20	285.90	278.40	9145.69	1.23	12197.99	4.37
O-C=O	297.20	287.10	278.40	2023.69	1.15	2530.39	0.91
Metal nitrides	409.20	397.37	391.40	6698.10	1.90	13811.86	3.18
C-N	409.20	400.38	391.40	3323.03	1.37	4916.82	1.14
Si_3N_4	409.20	398.78	391.40	1154.71	1.06	1323.51	0.31
Al_2O_3	544.20	530.26	524.40	82500.41	2.09	186939.43	27.66
Mental oxides	544.20	528.68	524.40	10425.38	1.29	14556.56	2.15
C=O	544.20	532.88	524.40	805.77	0.64	554.14	0.08

Supplementary Table 9. XPS narrow spectra date of the CBA@AAO.

Name	Start BE	Peak BE	End BE	Height CPS	FWHM eV	Area (P) CPS.eV	At. %
Metal carbide	296.50	282.51	277.70	63874.19	2.33	161172.13	54.77
C-C	296.50	284.80	277.70	12752.22	2.14	29498.56	10.04
Metal nitrides	408.50	396.91	390.70	8098.19	2.19	19198.38	4.20
C-N	408.50	399.58	390.70	3192.99	1.60	5522.91	1.21
Si_3N_4	408.50	398.38	390.70	726.76	0.45	348.35	0.08
Al_2O_3	543.50	529.66	523.70	84237.38	2.16	196764.28	27.65
Mental oxides	543.50	527.88	523.70	10488.53	1.29	14644.74	2.06

Supplementary Table 10. Pore diameter, density and porosity of five types of AAO.

AAO (nm)	Actual pore diameter (nm)	Mean pore diameter (nm)	Pore density ($pores/cm^2$)	Poriness (%)
20-30	20.5-38.1	31.0±3.4	2.8×10^{10}	36.2
40-70	48.1-88.3	60.3±7.0	8.8×10^{9}	17.5
800-100	51.0-123.6	82.3±9.3	7.3×10^{9}	22.2
110-150	79.0-135.6	101.4±9.7	4.4×10^{9}	40.4
200-300	135.3-392.2	293.4±48.9	4.4×10^{9}	61.8

Supplementary Table 11. Jackknifed classification matrix of 3 drugs. Based on DNA probe-N_CB_S, a jackknifed classification matrix was employed to differentiate different concentrations of cathinone, methcathinone and ethcathinone.

Jankknifned Classification Matrix of three drugs at different concentrations												
		Cathinone				Methcathinone			Ethcathinone			%correct
		Control	1 fM	0.1 nM	1 nM	Control	0.1 nM	1 nM	Control	0.1 nM	1 nM	
**Cathinone**	Control	**5**	0	0	0	0	0	0	0	0	0	100
	1 fM	0	**5**	0	0	0	0	0	0	0	0	100
	0.1 nM	0	0	**5**	0	0	0	0	0	0	0	100
	1 nM	0	0	0	**5**	0	0	0	0	0	0	100
**Methcathinone**	Control	0	0	0	0	**5**	0	0	0	0	0	100
	0.1 nM	0	0	0	0	2	**3**	0	0	0	0	60
	1 nM	0	0	0	0	0	0	**4**	0	1	0	80
**Ethcathinone**	Control	0	0	0	0	0	0	0	**5**	0	0	100
	0.1 nM	0	0	0	0	0	0	0	0	**5**	0	100
	1 nM	0	0	0	0	0	0	0	0	0	**5**	100
**Total**		5	5	5	5	7	3	4	5	6	5	**94**
**N = 50**				**N Correct = 47**				**Proportion Correct = 0.94**				

Supplementary Table 12. Jackknifed classification matrix of 14 drugs at identical concentrations.

Janckknifed Classification Matrix on CBA@AAO (Fourteen drugs at same concentration were detected in Tris-HCl buffer)															
	Cat	Met	Eth	Nor	Ket	Amp	MDMA	Phe	Coc	Her	Caf	Pro	Par	PSE	**%correct**
Cat	**5**	0	0	0	0	0	0	0	0	0	0	0	0	0	100
Met	0	**5**	0	0	0	0	0	0	0	0	0	0	0	0	100
Eth	0	0	**5**	0	0	0	0	0	0	0	0	0	0	0	100
Nor	0	0	0	**5**	0	0	0	0	0	0	0	0	0	0	100
Ket	0	0	0	0	**5**	0	0	0	0	0	0	0	0	0	100
Amp	0	0	0	0	0	**5**	0	0	0	0	0	0	0	0	100
MDMA	0	0	0	0	0	0	**5**	0	0	0	0	0	0	0	100
Phe	0	0	0	0	0	0	0	**5**	0	0	0	0	0	0	100
Coc	0	0	0	0	0	0	0	0	**5**	0	0	0	0	0	100
Her	0	0	0	0	0	0	0	0	0	**5**	0	0	0	0	100
Caf	0	0	0	0	0	0	0	0	0	0	**5**	0	0	0	100
Pro	0	0	0	0	0	0	0	0	0	0	0	**5**	0	0	100
Par	0	0	0	0	0	0	0	0	0	0	0	0	**5**	0	100
PSE	0	0	0	0	0	0	0	0	0	0	0	0	0	**5**	100
**Total**	5	5	5	5	5	5	5	5	5	5	5	5	5	5	**100**
**N = 70**					**N Correct = 70**					**Proportion Correct = 1.00**					

Supplementary Table 13. Jackknife classification matrix of 4 drugs and 2 disruptors in artificial sweat. Based on the co-calibration strategy, four drugs and two common sweat markers in artificial sweat were classified by jackknife classification matrix.

Janckknifed Classification Matrix on CBA&C4@AAO (Four drugs and two disruptors were detected in artificial sweat)							
	Cat	Met	Eth	Nor	Glu	Lac	**%correct**
Cat	**5**	0	0	0	0	0	100
Met	0	**5**	0	0	0	0	100
Eth	0	0	**5**	0	0	0	100
Nor	0	0	0	**5**	0	0	100
Glu	0	0	0	0	**5**	0	100
Lac	0	0	0	0	1	**4**	100
**Total**	5	5	5	5	6	4	**97**
**N = 30**					**N Correct = 29**		**Proportion Correct = 0.97**

Supplementary Table 14. Research progress on cathinone monitoring.

Detection method	Linear range	LOD (ng/mL)	Ref.
Electrochemical	1-10^4 nM	0.027	1
LC-MS/MS	0.22-2.31 ng/mL	0.07-0.69	2
MIP	2.0-12.0 μM	4.76	3
HPLC-MS/MS	7.46-149 ng/mL	0.002	4
AIE-MIP	2-12 μM	7.74	5
EKS-CE	15-250 ng/mL	4-8	6
CE	25-1000 ng/mL	15-45	7
GC-MS	2-50 ng/mL	1-10	8
MIF-SPE	1.5×10^{-5}-1.1×10^{-2} μg/mL	8.9×10^{-3}	9
LC-QQQ-MS/MS	1-10 ng/mL	0.01	10
CBA@AAO	1-10^4 fM	5.93×10^{-8}	
CBA&C4@AAO	0.1-10^7 fM	1.87×10^{-7}	**This work**
CBA&C4@AAO (In artificial sweat)	10^3-10^8 fM	5.34×10^{-7}	

4. Supplementary References

1. Zhang, X., Tang, Y., Wu, H. et al. Integrated aptasensor array for sweat drug analysis. *Anal. Chem.* **94**, 22, 7936-7943 (2022).

2. Hong, C., Yan, Y., Tu, T. et al. Efficient flat membrane based liquid phase microextraction of cathinones from environmental water and biological samples. *Talanta. Open.* **7**, 100232 (2023).

3. Hu, R., Yan, Y., Jiang, L. et al. Determination of total cathinones with a single molecularly imprinted fluorescent sensor assisted by electromembrane microextraction. *Microchim. Acta.* **189**, 324 (2022).

4. Han, C., Tan, D., Wang, Y. et al. Selective extraction of synthetic cathinones new psychoactive substances from wastewater, urine and cocktail using dummy molecularly imprinted polymers. *J. Pharm. Biomed. Anal.* **215**, 114765 (2022).

5. Yan, Y., Jiang, L., Zhang, S. et al. Specific "light-up" sensor made easy: An aggregation induced emission monomer for molecular imprinting. *Biosens. Bioelectron.* **205**, 114113 (2022).

6. Pérez-Alcaraz, A., Borrull, F., Aguilar, C. et al. An electrokinetic supercharging approach for the enantiodetermination of cathinones in urine samples by capillary electrophoresis. *Microchem. J.* **158**, 105300 (2020).

7. Pérez-Alcaraz, A., Borrull, F., Calull, M. et al. Field-amplified sample injection combined with CE for the enantiodetermination of cathinones in urine samples. *J. Sep. Sci.* **43**, 2914-2924 (2020).

8. Mercieca, G., Odoardi, S., Cassar, M. et al. Rapid and simple procedure for the determination of cathinones, amphetamine-like stimulants and other new psychoactive substances in blood and urine by GC-MS. *J. Pharm. Biomed. Anal.* **149**, 494-501 (2018).

9. Zang, D., Yan, M., Ge, S. et al. A disposable simultaneous electrochemical sensor array based on a molecularly imprinted film at a NH_2-graphene modified screen-printed electrode for determination of psychotropic drugs. *Analyst* **138**, 2704 (2013).

10. Swortwood, M. J., Boland, D. M. & DeCaprio, A. P. Determination of 32 cathinone derivatives and other designer drugs in serum by comprehensive LC-QQQ-MS/MS analysis. *Anal. Bioanal. Chem.* **405**, 1383-1397 (2013).
